# Specificity Effects of Amino Acid Substitutions in Promiscuous Hydrolases: Context‐Dependence of Catalytic Residue Contributions to Local Fitness Landscapes in Nearby Sequence Space

**DOI:** 10.1002/cbic.201600657

**Published:** 2017-05-02

**Authors:** Christopher D. Bayer, Bert van Loo, Florian Hollfelder

**Affiliations:** ^1^ Department of Biochemistry University of Cambridge 80 Tennis Court Road CB2 1GA Cambridge UK; ^2^ Present address: c-LEcta GmbH Perlickstrasse 5 04103 Leipzig Germany; ^3^ Present address: Institute for Evolution and Biodiversity University of Münster Hüfferstrasse 1 48149 Münster Germany

**Keywords:** catalytic promiscuity, fitness landscapes, molecular evolution, mutagenesis, phosphate transfer

## Abstract

Catalytic promiscuity can facilitate evolution of enzyme functions—a multifunctional catalyst may act as a springboard for efficient functional adaptation. We test the effect of single mutations on multiple activities in two groups of promiscuous AP superfamily members to probe this hypothesis. We quantify the effect of site‐saturating mutagenesis of an analogous, nucleophile‐flanking residue in two superfamily members: an arylsulfatase (AS) and a phosphonate monoester hydrolase (PMH). Statistical analysis suggests that no one physicochemical characteristic alone explains the mutational effects. Instead, these effects appear to be dominated by their structural context. Likewise, the effect of changing the catalytic nucleophile itself is not reaction‐type‐specific. Mapping of “fitness landscapes” of four activities onto the possible variation of a chosen sequence position revealed tremendous potential for respecialization of AP superfamily members through single‐point mutations, highlighting catalytic promiscuity as a powerful predictor of adaptive potential.

## Introduction

In apparent conflict with traditional views on enzymatic catalysis, promiscuous enzymes are not exclusively specific for single substrates, but turn over multiple substrates, even if those compounds differ substantially in their molecular recognition properties.^1^ The secondary, promiscuous substrates of such catalysts can differ either only in peripheral spectator groups that do not directly participate in the reaction (*substrate* promiscuity), or in groups that participate in the reaction, sometimes even through a substantially different mechanism (*catalytic* promiscuity).^1^, ^2^, ^3^, ^4^, ^5^, ^6^, ^7^, ^8^, ^9^, ^10^

Members of the alkaline phosphatase (AP) superfamily exhibit widespread catalytic promiscuity, catalyzing multiple, chemically distinct phosphate‐ and sulfate‐transfer reactions.^2^, ^11^, ^12^, ^13^, ^14^, ^15^, ^16^, ^17^ Crosswise promiscuity—the promiscuous activities of one enzyme are the primary activities of another—is commonplace between members of this superfamily.^2^, ^17^, ^18^ The chemical versatility of these catalysts raises fundamental questions about the molecular recognition mechanisms that these enzymes exploit in order to bind and turn over substrates that differ widely in charge, size, reactivity, and the identities of the bonds that are made and broken.^1^, ^19^

Catalytic promiscuity has been taken as an indication of a functional relationship between evolutionarily related enzymes, reflecting either evolutionary history or future potential.^8^, ^10^, ^20^, ^21^ A protein‐coding gene that is under selective pressure for a particular activity can serve, after gene duplication, as starting point for the evolution of a gene with a new function. The likelihood that one of these duplicated genes acquires a level of a new activity high enough to provide an immediate selective advantage is low. However, the presence of a promiscuous activity gives a “head start” to adaptation, because there are fewer steps in sequence space to travel until a new activity reaches a selective threshold.^10^ Any functionally beneficial mutation can carry a stability cost,^11^, ^22^, ^23^ thus dictating that the number of mutations must be minimized during a successful functional switch. Ideally, the level of promiscuous activity in the original protein should be high enough to provide a selective advantage immediately after gene duplication: that is, the enzyme must be able to accommodate considerable levels of catalysis for both substrates. Promiscuity of catalysts is thus implicit in the evolutionary model of Ohno,^24^ but also in the alternative scenarios of innovation/adaptation/divergence (IAD) and plasticity/relaxation/mutation (PRM).^25^, ^26^, ^27^

**Figure 1:**
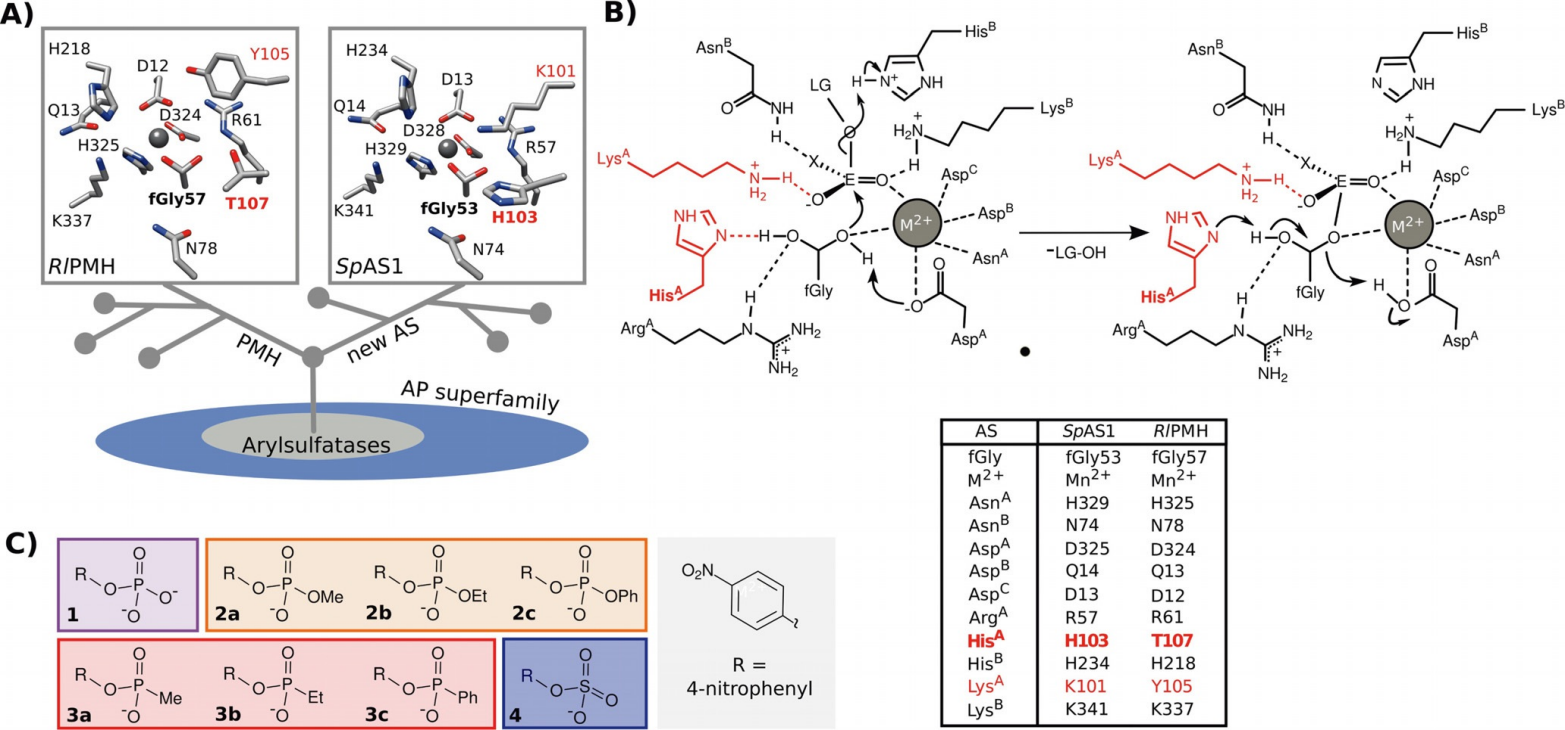
Active sites in the PMH and AS branches of the AP superfamily and the reactions catalyzed. A) Bottom: schematic representation of evolutionary relationships between PMHs and the new ASs that together form a group of enzymes related to arylsulfatases within the AP superfamily.^17^ Top: the conserved active sites of PMHs and ASs, represented by *Rl*PMH (PDB ID: 2VQR) and *Sp*AS1 (PDB ID: 4UPI). Both enzyme classes contain the same fGly nucleophile, arising from co‐translational modification of a conserved cysteine residue). PMHs and the new ASs differ in two active‐site positions: K101 and H103 of *Sp*AS1 and Y105 and T107 in *Rl*PMH (red labels in both structures). The residue structurally analogous to H103/T107 is a conserved histidine^90^ (His^A^ according to the nomenclature of Hanson et al.^35^) in all known AP‐superfamily arylsulfatases.^35^ In the majority of PMHs, the corresponding residue is an aspartate moiety, but a group of PMHs with a threonine residue at this position, such as *Rl*PMH, are the most proficient PMHs currently known.^17^ B) Mechanism of new ASs.^11^, ^17^ Residue identifiers follow the revised Hanson nomenclature.^17^, ^35^ “E” is an electrophile (either P or S), and “X” is variously oxygen (sulfate monoester and phosphate monoester), an alkyl or aryl group (phosphonate monoesters), or an alkoxy or phenoxy group (phosphate diesters). LG denotes the leaving group (here 4‐nitrophenyl). Most residues are analogous in ASs and PMHs. For example, Asp^B^ is a glutamine residue and Asn^A^ a histidine residue both in PMHs and in “new” ASs such as *Sp*AS1. Asn^B^ is not universally conserved among ASs but is 100 % conserved in PMHs and new ASs.^17^ Lys^A^ is a tyrosine residue in PMHs, so its possible charge–charge interactions with the substrate are not available. No unambiguous function could be assigned to the aspartate residue found in D‐PMHs and the threonine residue found in T‐PMHs (such as *Rl*PMH) at the His^A^ position. However, T107 in *Rl*PMH appears to coordinate the fGly nucleophile through a hydrogen bond.^11^ C) Structures of phospho‐ (compounds **1**–**3**) and sulfoester (compounds **4**) substrates used in this study. The background color‐coding is used to identify the reactions of the various substrates in subsequent figures.

Comparative studies of functional divergence within promiscuous enzyme families have been used successfully to provide insight into the evolution of function in various protein families.^28^, ^29^, ^30^, ^31^, ^32^ Within the AP superfamily, two structurally and phylogenetically related, but functionally divergent groups of arylsulfatases (ASs) and phosphonate monoester hydrolases (PMHs, Figure 1 A),^11^, ^14^, ^17^ provide an opportunity to explore pathways for their functional interconversion. The amino acid sequence conservation amongst the branches of PMHs and the “new” ASs^17^ is moderate (31–34 %), but their structures align well (r.m.s.d. 1.62–1.85 Å)^17^ and appear highly conserved. Despite the low overall sequence homology, most active‐site residues suggested^17^ to assist in substrate binding, nucleophilic attack, or leaving group stabilization are conserved in both ASs and PMHs (Figure 1 A). Their catalytic mechanisms (Figure 1 B) involve nucleophilic catalysis (by a formylglycine (fGly) residue), presumably assisted by general base catalysis, to pass through a transition state (TS) in which negative charge development on the leaving group and phosphoryl oxygen atoms is stabilized by cationic groups (that is, Lewis acid catalysis) or general acid catalysis.^33^ The resulting hemiacetal intermediate needs to be cleaved to regenerate the nucleophile. In ASs, a histidine (His^A^ in Figure 1 B) residue adjacent to the fGly nucleophile has been postulated to assist in the breakdown of the intermediate through general base catalysis.^34^, ^35^ In contrast, the threonine residue present at the “His^A^”‐position in *Rhizobium leguminosarum* PMH (*Rl*PMH) is unlikely to act as a general base and consequently its mutation into an alanine residue had no detrimental effect on the enzyme‐catalyzed conversion of phosphonate monoester **3 c**.^11^

The substrates hydrolyzed by members of the AP superfamily are diverse in their molecular recognition properties: members of this superfamily have been shown to convert substrates with one, two, or no negative charges, and the substrates have half‐lives from several months to millions of years,^2^, ^6^, ^11^, ^12^, ^13^, ^14^, ^15^, ^16^, ^17^, ^18^ thus implying very different catalytic requirements. This chemical diversity is further supported by the observation that the hydrolytic reactions of phosphate diesters and phosphonate monoesters proceed via concerted TSs,^36^, ^37^ whereas those of phosphate and sulfate monoesters are characterized by more expanded, dissociative TSs.^37^, ^38^, ^39^, ^40^

Here we report the effects of active‐site mutations on catalytic rates for multiple substrates for several of the related ASs and PMHs mentioned above. The resulting systematic analysis of the effects of residue substitutions in homologous positions on catalysis of the diverse chemical reactions in the AP superfamily probes the feasibility of establishing new activities during adaptation, and illustrates how structural constraints of the protein framework shape the effect of mutations on primary and promiscuous activities. Previous studies of protein mutagenesis and evolution have highlighted the importance of active‐site and adjacent residues for enzyme function.^41^, ^42^, ^43^ Our approach targets residues that form part of the active site or are in close contact with active‐site residues. Kinetic data for mutants were measured, and the data were subjected to statistical analyses to study relationships between mutations and the observed rates as a function of chemical properties of substrates or reaction types (e.g., substrate charge, nature of TSs).

## Results and Discussion

### Mapping local fitness landscapes: Site‐saturation mutagenesis of structurally analogous active‐site positions in PMH and AS families

We investigated the accessibility of improvements of promiscuous activities by single amino acid replacement mutagenesis of an analogous position (His^A^ in Figure 1 B) in one representative each of the AS (*Sp*AS1) and PMH (*Rl*PMH) families. This residue is located within hydrogen‐bonding distance of the fGly nucleophile,^11^, ^14^, ^17^, ^34^ and the nature of this residue can be used to differentiate between ASs (His) and PMHs (Asp/Thr) (that is, H103 in *Sp*AS1 and T107 in *Rl*PMH).

A full set of wild‐type and all 19 standard proteinogenic amino acid substitutions was generated, resulting in two sets of variants for *Sp*AS1 (H103X) and *Rl*PMH (T107X), expressed in *Escherichia coli* and isolated from crude lysate by Strep‐tag based affinity purification (for details see the Experimental Section and Figure S1 in the Supporting Information). Each variant was tested for activity towards each of the four substrate classes hydrolyzed by ASs and PMHs (phosphate monoester **1**, phosphate diester **2 b**, phosphonate monoester **3 a**, and sulfate monoester **4**, Figure 1 C). Activity tests were performed at substrate concentrations approaching first‐order conditions with regard to substrate (i.e., at substrate concentrations at least two to three times lower than the corresponding wild‐type Michaelis constant, *K*_M_). Under these conditions, differences in observed reaction rate are representative of changes in catalytic efficiency (*k*_cat_/*K*_M_; Experimental Section, Figure S2, Table S2). These enzyme activity measurements were corrected for variations in protein concentration (relative to the wild type). As a consequence, they can be directly interpreted as the ratio of second‐order rate constants, kmutant2 /kWT2 . This ratio provides a comparison of the first irreversible steps of the reaction sequence, in this case the formation of the fGly intermediate.

**Figure 2:**
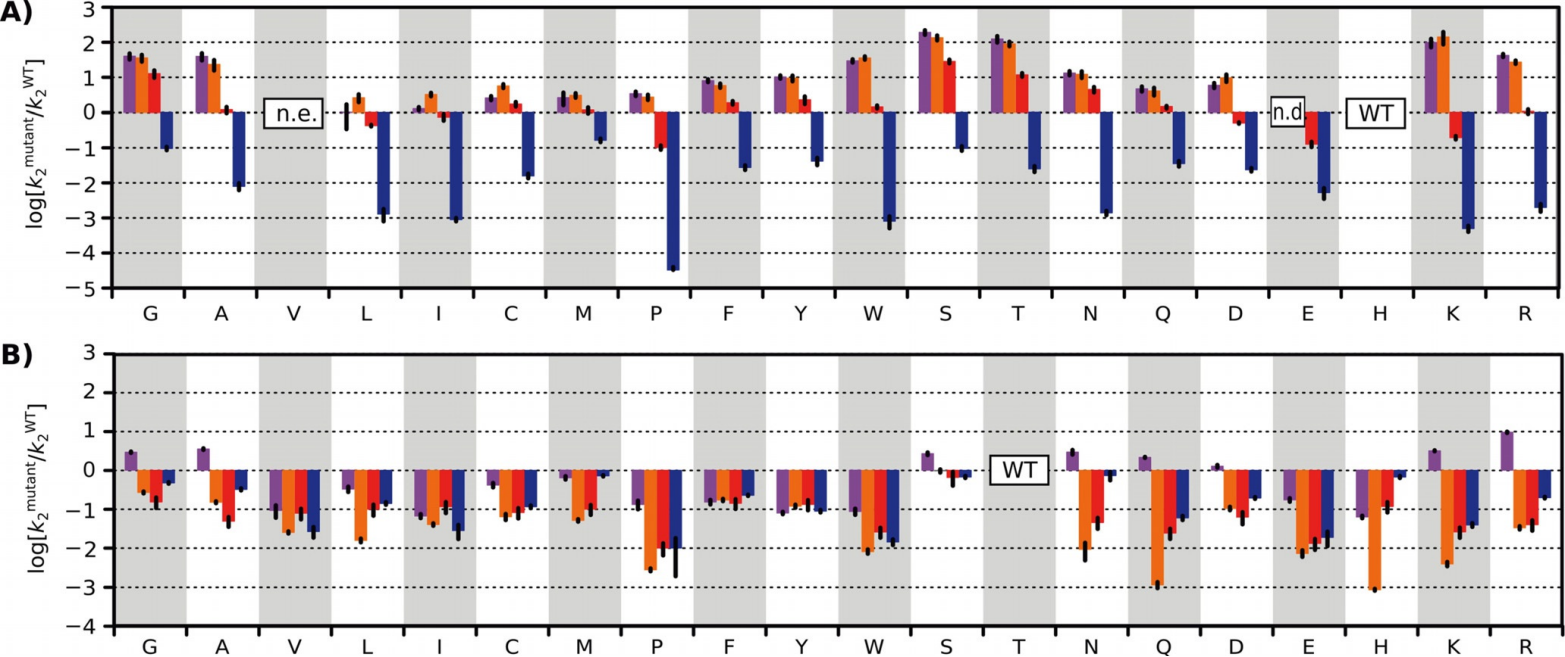
The effect of mutation of A) H103 in SpAS1, and B) T107 in RlPMH on the enzyme‐catalyzed hydrolysis of phosphate monoester **1** (purple), phosphate diester **2** **b** (orange), phosphonate monoester **3** **a** (red), and sulfate monoester **4** (blue). Initial rates (*V*_0_) were measured at substrate concentrations at least two times lower than the wild‐type *K*_M_, at which point changes in the observed second‐order rate constant (*k*_2_=*v*_0_/[enzyme]; i.e., initial rates normalized for varying protein concentrations) translate directly into changes in *k*_cat_/*K*_M_ (Figure S2 and Table S2). Changes in enzyme activities are indicated relative to wild type (log(kmutant2 /kWT2 )). The box labelled “n.d.” indicates that no significant activity above background was observed (on the basis of the detection limit, activity would be at least ≈80 times (phosphate monoester) or ≈60 times (phosphate diester) lower than wild type). Mutant H103V could not be expressed (n.e.). All rates were determined in triplicate at 30 °C in 100 mm Tris**⋅**HCl, 500 mm NaCl, and 100 μm MnCl_2_ at pH 7.5. Error bars represent the standard deviations of measurements. See Tables S3 (SpAS1 H103X) and S4 (RlPMH T107X) for details.

In *Sp*AS1, mutation of H103 into any other residue resulted in a more than tenfold (for ten out of 19 substitutions, >100‐fold) decrease in the primary activity (hydrolysis of sulfate monoester **4**, Figure 2 A, Table S3), thus confirming that a histidine residue at this position is the optimal residue for the native activity of ASs. At the same time, activity toward phosphate mono‐ and diesters increased for all mutants, with more than tenfold improvement observed for ten out of 19 mutants (Figure 2 B, Table S3). Some of the mutants even show a ≈100‐fold increase in those activities (observed for mutants H103S, ‐T, and ‐K). Changes in enzyme‐catalyzed phosphonate monoester hydrolysis ranged from a 30‐fold increase to a tenfold decrease in catalytic efficiency, and mutants with strongly improved phosphomono‐ and ‐diesterase activities showed decreased phosphonate monoesterase activity (e.g., H103K, ≈100‐fold increase vs. ≈tenfold decrease, respectively).

**Figure 3:**
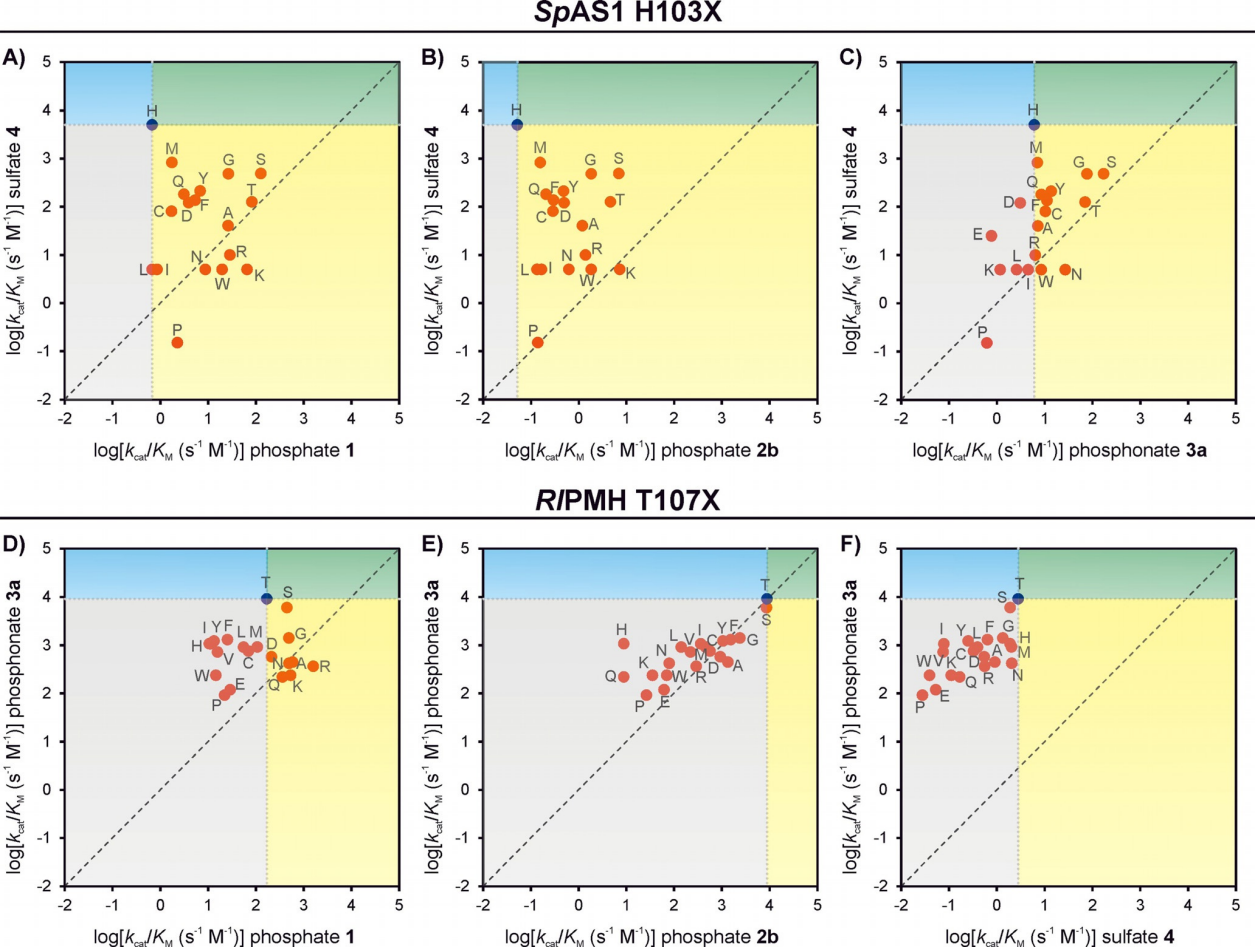
Trade‐off landscapes illustrating the effect of mutation of *Sp*AS1 H103X and *Rl*PMH T107X, shown as plots of the catalytic efficiencies (*k*_cat_/*K*_M_) toward the primary substrate versus those of each of the promiscuous substrates. The mutations are grouped into the following categories: 1) improved specialists (blue quadrant, increased specificity for the cognate activity), 2) enzymes improved only in their promiscuous activity (yellow), 3) general improvers (green, all activities increased), and 4) general decreasers (gray, mutation affects both activities detrimentally). The diagonal represents the locations of hypothetical generalists, which catalyze both reactions at equal rates. None of the mutations resulted in an increase in the primary activity (in which case they would have been observed in the green or blue quadrants). Catalytic efficiencies (*k*_cat_/*K*_M_) for the various mutants were calculated from wild‐type levels (as listed in Tables S9 (*Sp*AS1) and S13 (*Rl*PMH)) by using the experimentally determined kmutant2 /kWT2 ratios for each substrate.

The differential effects of the various mutations, resulting in up to 10^5^‐fold changes in specificity between substrate pairs, confirm the proposed role of the nucleophile‐flanking residue H103 as a specificity determinant between ASs and PMHs. Five out of the 19 possible mutants no longer have sulfate monoester hydrolysis as their “best” activity (Figure 3 A–C). This effect is particularly prominent for mutation H103K, which switches the enzyme from a ≈10^4^‐fold preference toward sulfate monoester **4** over phosphate monoester **1** to a tenfold preference in the reverse direction, accompanied by a 100‐fold improvement towards phosphate monoester **1**.

The results with the library *Sp*AS1 H103X are reminiscent of those obtained in the study of a site‐saturation library of E192 in *N*‐acetylneuraminic acid lyase,^44^ for which all substitutions lead to a reduction in its native retro‐aldol cleavage activity, accompanied by an increase in activity toward one of its promiscuous substrates for 16 of 19 possible mutations. The results for both enzymes reinforce the notion that promiscuous reactions, which do not use the active‐site functionalities in an optimized way, might be more robust towards (or even more readily improved by) a small number of mutations than the native activity. In both cases, the new activity was improved and the original one had decreased, thus causing a large specificity switch, as was also observed in several other studies in which specificity changes of promiscuous enzymes were caused by combinations of beneficial effects on one activity and detrimental effects on another.^7^, ^21^, ^45^, ^46^ The drastic changes in activity and specificity observed for the H103X mutants further add to a substantial body of evidence that shows that the chemical function and specificity of an enzyme can be changed with only a few amino acid substitutions.^41^, ^43^, ^47^

Unlike in *Sp*AS1, substitutions in the analogous position T107 in *Rl*PMH predominantly result in decreased catalytic activities toward *all four* substrate classes, with the exception of phosphomonoesterase activity, for which eight out of 19 mutants show improvements (largest increase: ≈tenfold in *Rl*PMH T107R, Figure 2 B and Table S4). However, even in the absence of substantial improvements, *Rl*PMH is robust to mutagenesis at T107: 10 out of 19 substitutions are “neutral” (<10‐fold decrease in catalytic efficiency) with respect to the two primary activities (in two of 19 cases for phosphodiesterase; in eight of 19 for phosphonate monoesterase). The various mutations in T107 in *Rl*PMH result in up to 700‐fold changes in substrate preference (e.g., for T107K, phosphomonoesterase vs. phosphodiesterase activities; Figures 2 and S10). However, the general specificity of, for example, the activity towards phosphonate monoester **3 a** over the promiscuous activity towards sulfate **4** is only reduced by less than tenfold by any of the mutations to T107; this stands out in contrast with the much larger effects found for *Sp*AS1 H103X. At least for a single‐residue mutation step in position 103/107, the observed readily accessible specificity change appears to be unidirectional (e.g., for *Sp*AS1 H103T). In contrast, the analogous “reverse” mutation T107H would not be an accessible first step in evolution starting from *Rl*PMH.

### Apparent redundancy of the active sites opens up multiple pathways for future adaptive evolution

Our results show that mutations affect promiscuous activities differentially, resulting in significant changes in specificities. In some cases they can even change which substrate is preferred (Figure 3). In *Sp*AS1 this switch occurs as a result of the introduction of one of five amino acids with very different molecular recognition features (P, W, N, K, and R). In particular for phosphate mono‐ and diester hydrolysis, mutation of H103 in *Sp*AS1 is beneficial in all cases, irrespective of which residue it is replaced with. There are no obvious amino acid properties that correlate with the degree of improvement (Figures S3 and S5): for example, both removal of the histidine side chain (H103G, H103A) or replacement with a large charged residue (H103K and H103R) show similar degrees of improvement in phosphomono‐ and ‐diesterase activity (Figures 2 A and 3 A, B). For phosphomonoesterase activity the results for H103K and H103R can be explained in terms of improved interaction of the now cationic side chain with the negative charge in the more charged substrates (and also the TS of phosphoryl transfer).^48^ However, the beneficial effect of H103G and H103A cannot be readily explained. The improvements for phosphomono‐ and ‐diesterase activity are positively correlated for *Sp*AS1 H103X (Figure S9, see below for details), which suggests that the difference in net charge between the primary and promiscuous substrates is not the reason for the differential effects of the mutation on sulfatase and phosphomonoesterase activity. These observations imply that a histidine residue in the conserved position is *disfavoring* phosphoryl transfer in the *Sp*AS1 active site, in which case its mutation into any other residue removes a limitation, rather than the chemical properties of the “new” side chain providing explicitly beneficial interactions for phosphoryl transfer.

**Figure 4:**
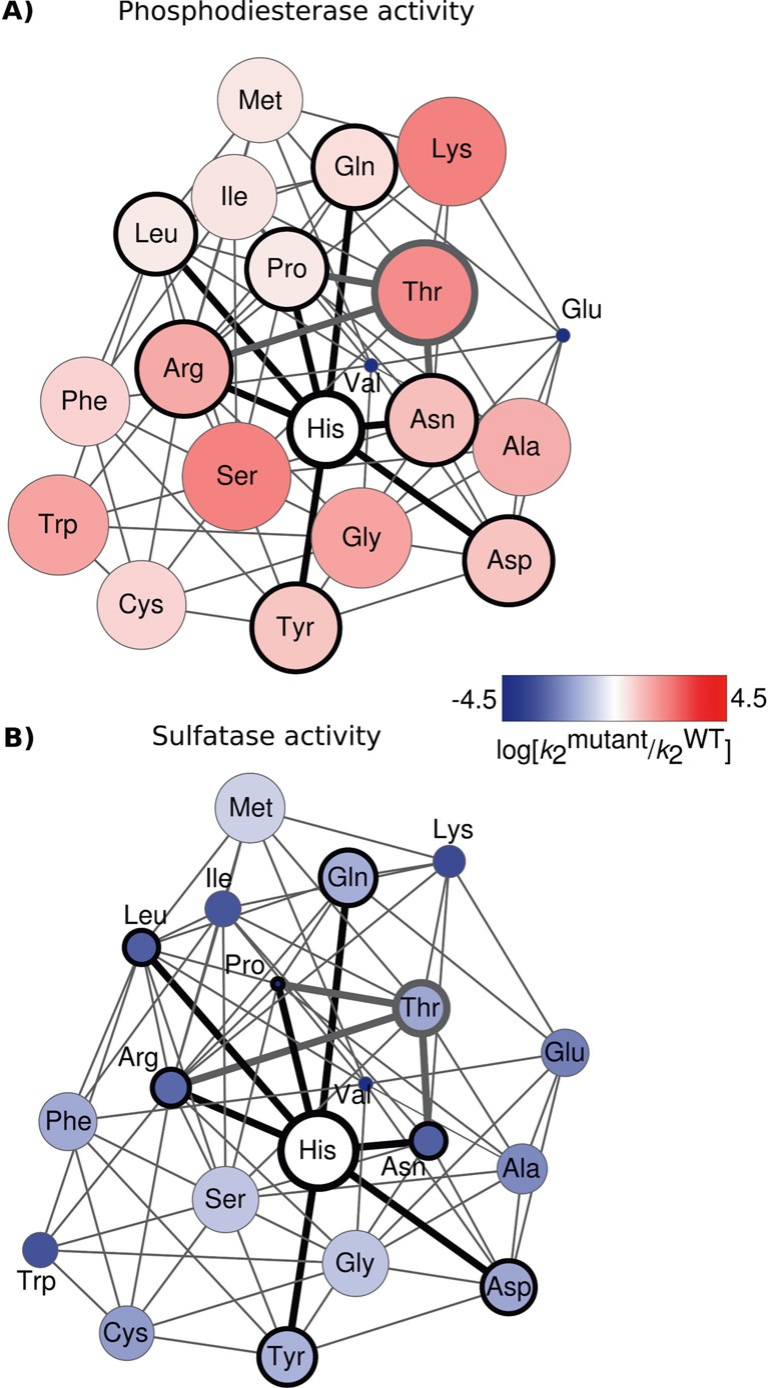
Local fitness landscapes for position 103 of *Sp*AS1: A) for its promiscuous phosphodiesterase activity, and B) for its primary sulfatase activity, represented as networks. Nodes (circles) correspond to the amino acid in position 103 in a given variant. Nodes are color‐coded according to the activity of a given variant relative to *Sp*AS1 WT as log [kmutant2 /kWT2 ] (see also Figure 2 A and Table S3). The node diameter was scaled according to log [kmutant2 /kWT2 ]+1, so that nodes bigger than His (WT) denote more active enzymes, whereas nodes smaller than His correspond to less‐active mutants. Edges (or connections) are only allowed between amino acids that can be interconverted without passing through a codon coding for a third amino acid (i.e., they can be converted either by a single nonsynonymous nucleotide substitution, or by one or two synonymous mutations followed by a nonsynonymous one). The wild‐type residue of *Sp*AS1 (His103) and all amino acids directly accessible from any of the histidine codons are highlighted in bold black. Subsequent steps that would lead to threonine, the amino acid of *Rl*PMH WT at position 107 (the “target” in a hypothetical evolutionary “trajectory”) are highlighted in bold gray. Thus, the black/gray subnetwork shows hypothetical trajectories of interconversion between histidine and threonine at position 103 of *Sp*AS1.

The site‐saturation mutagenesis data obtained in this study were used to build a network of mutational transitions that corresponds to an empirical fitness landscape.^49^, ^50^, ^51^, ^52^, ^89^ Local fitness landscapes were constructed for mutations in the nucleophile flanking residue in *Sp*AS1 (Figure 4) and *Rl*PMH (Figure S8). We refer to these landscapes as “local” because we map only the immediate vicinity of the wild type, resulting from a single‐residue mutation. This approach is similar to previous studies on constraints for in vitro protein evolution.^49^, ^52^, ^53^, ^54^, ^55^ For *Sp*AS1 H103X, for example, the fitness landscapes for the primary sulfatase and the promiscuous phosphodiesterase activities show a steep decline and a steep incline, respectively, in multiple directions (Figure 4). For the primary sulfatase activity, all amino acids that are directly accessible from any codon coding for the wild‐type residue (His) result in a more than tenfold reduction in activity. In fact, all amino acids other than the wild‐type residue (His) exhibit lower sulfatase activity. In evolutionary terms, *Sp*AS1 WT is at the global fitness peak for its primary activity with respect to position 103. For its promiscuous activities, the situation is quite the opposite: for example, for its promiscuous phosphodiesterase activity, *Sp*AS1 WT is almost at the global fitness minimum for position 103 (Figure 4 A), with the exception of the mutants H103E (detrimental to all four activities) and H103V (no expression). More importantly, His103 is located directly in a fitness “well”: that is, any of the direct neighbors accessible from histidine‐encoding codons through a single‐nucleotide substitution are better phosphodiesterases than the wild type. This suggests that once *Sp*AS1 is under selective pressure for improved phosphodiesterase activity, *any* non‐synonymous mutation in the codon for His103 is beneficial and can remain in the gene pool, thus providing multiple starting points for adaptive evolution. From three of the mutations accessible by single‐nucleotide substitution from *Sp*AS1 H103 (wild type)—arginine, proline, and asparagine—the codon for threonine is accessible through just one additional nucleotide change. This suggests that, in *Sp*AS1, the transition from the sulfatase residue His^A^ to the signature residue of proficient phosphodiesterases/phosphonate monoester hydrolases, threonine, is accessible by a pathway via two subsequent nucleotide exchanges. This means that an adaptive pathway from sulfatase to phosphatase that does not require mutations elsewhere in the protein exists.

The strongly detrimental effect of any mutation on the primary sulfatase activity rules out a scenario in which a strong increase in a promiscuous activity is accompanied by a relatively small change in the original function (i.e., maintained at >10 % of the wild‐type level), which has been reported for the “generalist intermediate scenario” characterized by low initial negative trade‐off.^7^, ^21^, ^56^ This observation is also more consistent with Ohno's rather than the IAD or PRM models.^24^, ^25^, ^26^, ^27^ The unavailability of this scenario suggests that mutations at this position would be unlikely to occur in vivo if the selection pressure for the original function were maintained: that is, duplication of the *Sp*AS1‐encoding gene would have to precede these mutations. Once the original selection pressure is relieved, there are multiple mutational paths that result in ten‐ to 100‐fold improvements in, for example, phosphomonoesterase (Figure 2 A) or phosphodiesterase activity (Figures 2 A and 4 A). Out of 19 substitutions of His103 with another amino acid, ten result in at least tenfold improvement of promiscuous phosphodi‐ or phosphomonoesterase activities (Figure 2 A), with four of them (Y, N, D, R) being accessible from histidine‐encoding codons through a single base pair substitution (Figure 4 A) and thus evolutionarily directly convertible.

This variety of possible amino acid substitutions opens up diverse pathways for further adaptive evolution. For example, the mutations H103G, H103T, and H103K—to a hydrophobic, a hydrophilic, and a positively charged residue, respectively—all result in >30‐fold improvements of both phosphomonoesterase and phosphodiesterase activities, but each of these substitutions would be expected to affect the electrostatic environment of the active site differently. Even the substitutions directly accessible from histidine‐encoding codons (H103Y, H103N, H103D, and H103R) are vastly different with respect to the expected effect their introduction will have on the physicochemical properties of the active site. The fact that multiple pathways result in improvement of a promiscuous activity, such as described in this study and elsewhere,^44^, ^53^, ^54^ provides evidence that a group of mutants at this position can act as “molecular quasi‐species”: that is, a group of variants of a protein sequence that are close in functional space (for the new activity), but have started to diverge in sequence space.^57^ Such populations of variants have been considered to be the real subject of selection (rather than single‐enzyme variants) and might, due to their higher diversity, increase the success of further evolution because they would reduce the likelihood of the occurrence of evolutionary dead‐ends.^57^, ^58^

**Figure 5:**
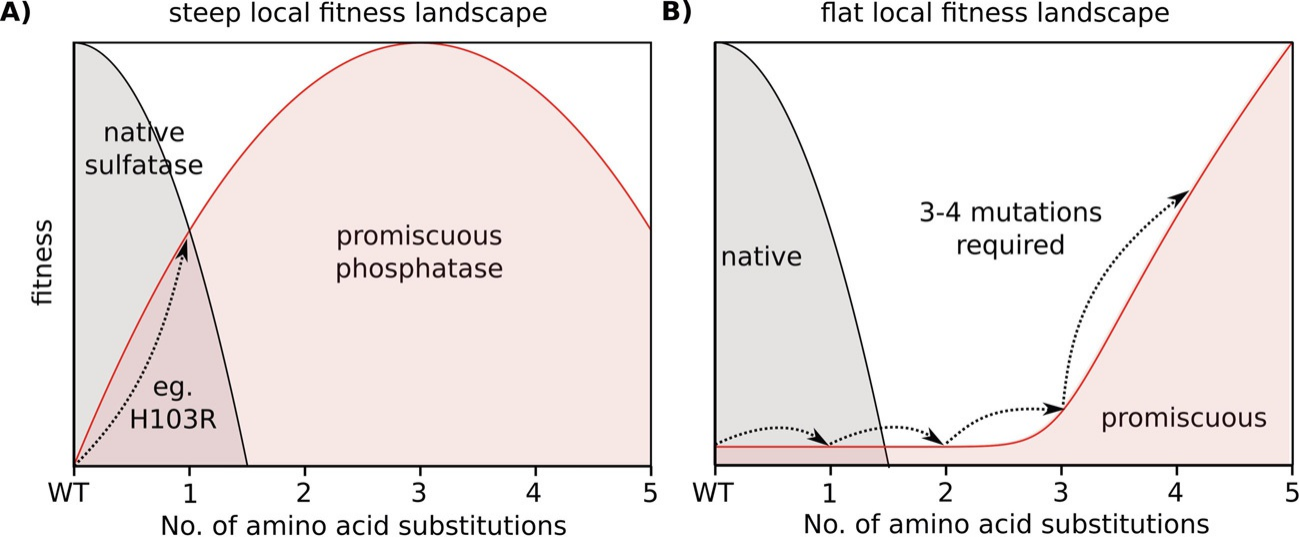
Illustration of evolutionary scenarios: overlap and steepness of fitness landscapes. Comparison of A) steep, and B) flat local fitness landscapes and the influence of the topology of the landscape on the accessibility of new functions. Grey: fitness landscapes of the main activity of a given wild‐type enzyme. Red: fitness landscapes of promiscuous activities. The sequence space is reduced to a single dimension (Hamming distance for the expressed amino acid sequence) for purposes of illustration. A) A scenario for rapid adaptation. The local fitness landscape for the promiscuous phosphatase activity of SpAS1 is apparently very steep, from our results of mutating residue H103: improvements of up to 100‐fold are already accessible through various single mutations, such as H103R (Figures 2 and 4). This means that the promiscuous function can be improved quickly to the level of a “generalist” that can hardly differentiate between primary and promiscuous activity. B) A scenario for adaptation requiring multiple mutations. The local fitness landscape for a new activity is very flat, with the consequence that three amino acid substitutions are required to reach significant improvements, such as the one reported by Sandström et al.,^59^ and four mutations are required to achieve an improvement that surpasses that illustrated for the case of a steep gradient in a fitness landscape, as displayed in (A).

The steep inclines observed in the fitness landscape for enzyme‐catalyzed phosphate monoester and phosphate diester hydrolysis (Figure 4 A) contrast with previous findings with other enzymes, in which combinations of mutations were required for substantial improvements in, for example, enantioselectivity.^59^ This was attributed to a very flat local fitness landscape (Figure 5 B), in which single mutations did not result in any significant improvement of the desired functionality. When multiple mutations are required, the chance that all of them arise is low. “Epistatic ratchets” can lower this likelihood even further:^60^ that is, the mutations can only be acquired in a specific order, limiting the number of possible evolutionary paths.^50^, ^52^, ^61^, ^62^ Because about one in three mutations in a protein is expected to be detrimental to function and/or stability,^9^, ^22^, ^23^ it is vital that beneficial levels of new activities are accessible through as few mutations as possible. Enzyme promiscuity has been suggested as a possible mechanism of such quick adaptation,^7^, ^10^, ^20^ because the presence of a promiscuous activity close to the selective threshold probably decreases the number of mutations needed to provide a selective advantage. Indeed, for *Sp*AS1, the observation of a promiscuous activity in the wild type would correctly predict rapid improvement in, for example, phosphomonoesterase activity through few mutations (or in fact, as observed, a single‐point mutation).

### Dependence of functional trade‐offs on physicochemical properties of substrates in different active‐site contexts

Conventional molecular recognition analysis—based on, for example, size or hydrophobicity of the various amino acid side chains (as in Figures S3–S6)—does not yield straightforward correlations. Similarity in substrate properties (charge, size) does not correlate with similar effects of identical mutations. The multidimensionality of the activity space (four substrates) further complicates the observed structure–function correlations between the various mutants. We therefore reduced the number of dimensions in activity space by use of principal component analysis (PCA),^63^ in order to detect and to visualize trends and correlations that are beyond the obvious, as has been done previously for analysis of the distributions of protein families or mutant proteins in promiscuous “activity spaces”.^57^, ^64^, ^65^, ^66^, ^67^

**Figure 6:**
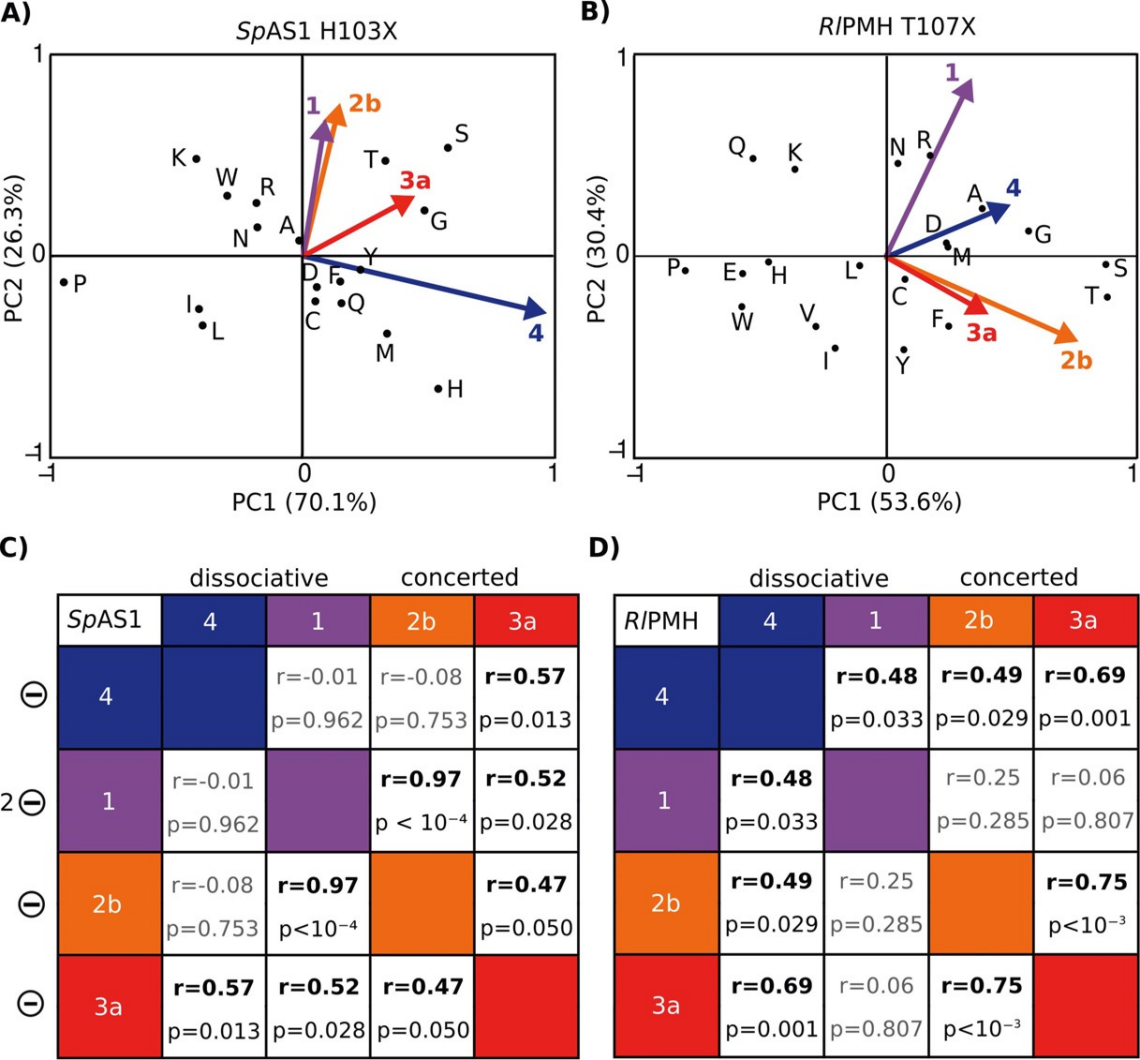
Principal component and correlation analyses of the effects of mutation of the nucleophile‐flanking residue in *Sp*AS1 (H103, panels A/C) and *Rl*PMH (T107, panels B/D). A), B) The original datasets for the effect of the various mutations in the nucleophile‐flanking residue (log [kmutant2 /kWT2 ], Figure 2, Tables S3 and S4) were subjected to principal component analysis to project the four‐dimensional activity space of substrates **1**, **2 b**, **3 a**, and **4** (Figure 1 C) onto a two‐dimensional “activity plane” defined by PC1 and PC2. PC1 and PC2 are linear combinations of the original dimensions of the dataset [generated from Eq. (1)], normalized to their own maximum values. PC1 and PC2 together describe >80 % of the variance in the original dataset for both enzymes (96 % for *Sp*AS1 H103X, 86 % for *Rl*PMH T107X). Mutants that are in proximity to each other in the plane defined by PC1 and PC2 have similar specificity profiles. The original dimensions (i.e., changes in activities on sulfate monoester **4** (blue), phosphonate monoester **3 a** (red), phosphate monoester **1** (purple) and phosphate diester **2 b** (orange)) are projected onto the coordinate system defined by PC1 and PC2 as colored vectors. In this plane activity increases for each activity are identified by the corresponding vectors for substrates **1**–**4** (panels A and B). Similarity of the vectors in direction and length indicates a correlation. C), D) Correlation analysis on the raw dataset prior to PCA. Correlation coefficients (*r*) are shown in boldface when the correlation is significant (*p*≤0.05); nonsignificant cases are shown in pale. Substrate charge (left) and nature of the transition state (TS) of the solution reaction (top) are indicated. The corresponding correlation plots are shown in Figure S9 (*Sp*AS1 H103X) and S10 (*Rl*PMH T017X).

PCA was performed on the set of mutants of each enzyme individually, and the coordinate system was calculated for AS and PMH independently. Because the coordinate system for each enzyme is optimized on the basis of the redundancy of the original dataset, the coordinate systems are not interchangeable between *Sp*AS1 H103X (Figure 6 A) and *Rl*PMH T107X (Figure 6 B). However, the relative positions of residue coordinates to each other, and their clustering, can be compared.

The distribution patterns of amino acid substitutions differ considerably between the two enzymes. Physicochemically or sterically very different residues are observed close together in activity space: for example, the cluster of C/D/F/Y/Q in *Sp*AS1 (Figure 6 A) and D/M or E/H in *Rl*PMH (Figure 6 B). At the same time, residues with similar catalytic groups or steric demands can be quite far apart: for example, Q/N or H/K/R in *Sp*AS1 and E/D or A/V in *Rl*PMH. In some cases, such as S/T, the proximity of mutations is conserved in both enzymes, but it is evident that the structural differences between the protein structures and the direct environments of the active sites must lead to quite different effects of identical amino acid substitutions.

The patterns of possible correlations between the effects of the various mutations on the four activities in the two enzymes differ considerably (Figure 6). For *Sp*AS1, the projected vectors for phosphate monoester **1** and phosphodiester **2 b** (Figure 6 A) are similar in both direction and length, thus indicating a positive correlation between the mutational effects in H103 on these two activities. This correlation was shown to be significant by direct comparison of these effects (*r*=0.97, *p*<10^−4^, Figures 6 C and S9). The correlation between phosphonate monoesterase activity (substrate **3 a**) and the other two phosphohydrolase activities is weaker (*r*=0.47 and 0.52), but still significant (*p*=0.050 and 0.028). The effects of the mutations on sulfate monoesterase activity were weakly, but significantly, correlated with those for phosphonate monoester **3 a** hydrolysis (*r*=0.57, *p*=0.0013). They showed no correlation with the effects on the other two phosphohydrolase activities. The physicochemical property that at least partly predicts these correlations is the nature of the electrophilic reaction center: data for all reactions involving phosphorus centers are correlated. In contrast, the TS of the solution reaction and the ground state (GS) charge of the substrate are poor predictors for correlation—the two activities that show the strongest correlation, phosphomonoesterase and phosphodiesterase, differ in both of these aspects (dissociative vs. associative and −2 vs. −1, respectively).^37^, ^39^, ^40^

The analysis for the effects of mutations in T107 in *Rl*PMH suggests a correlation that is governed by the nature of the TS in solution, because the projected vectors both of the phosphodiesterase/phosphonate monoesterase (concerted) and of the phosphatase/sulfatase (dissociative) pairs appear in the same quadrant of the PCA (Figure 6 B). Direct comparison of both substrate pairs confirmed this observation (Figures 6 D and S10). The same direct comparisons between all possible substrate pairs also indicated that substrate charge at least partly predicts correlation, because all reactions with a substrate charge of −1 (at the experimental pH of 7.5) are correlated, and the correlation between the two dissociative reactions, which differ in GS charge, is weaker than that between the two concerted reactions that have the same substrate charge (*r*=0.48; *p*=0.033 vs. *r*=0.75; *p*=2×10^−4^).

Comparison of the possible physicochemical predictors for the effect of mutations in the nucleophile flanking residue for both enzymes studied here shows that their predictive values are most likely strongly context dependent. Furthermore, even within one enzyme, none of the three properties described thus far—GS charge, TS of the solution reaction, or reaction center—is strongly dominating, thus suggesting that a more complex interplay of various factors governs the trade‐off between different activities.

**Figure 7:**
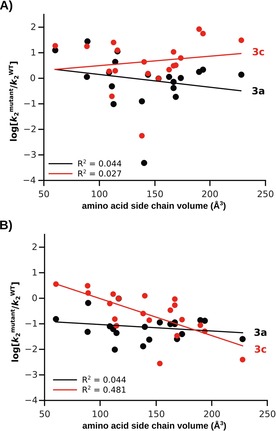
Dependence of mutational effects (log [kmutant2 /kWT2 ]) on residue volume of A) *Sp*AS1 H103X, and B) *Rl*PMH T107X. Volumes of amino acid side chains were taken from Zamyatin et al.^88^ (See Table S5 for details). Black: 4‐nitrophenyl methylphosphonate (**3 a**). Red: 4‐nitrophenyl phenylphosphonate (**3 c**). Linear regression lines are shown in the plots. A) *Sp*AS1 H103. Correlation coefficient *R* ^2^ for methylphosphonate **3 a** (black): 0.044. *R* ^2^ for phenylphosphonate **3 c**: 0.027. B) *Rl*PMH T107. Correlation coefficient *R* ^2^ for methylphosphonate **3 a** (black): 0.044. *R* ^2^ for phenylphosphonate **3 c**: 0.481. Please refer to Figures S3–S6 for a more detailed analysis of the interplay of substituted residue volume/hydrophobicity and activity. Steric effects are further analyzed in Figures S11 and S12, with the focus on how the activity of the bulkiest substrate, phosphonate **3 c**, is correlated to smaller substrates.

Inclusion of specificity data for all variants of both enzymes for a bulky substrate (Figures 7, S11, and S12) suggests that the three properties discussed above are all far less important than the size of the substrate in question. Activity toward phosphonate monoester **3 c** shows no correlation with that toward the less bulky, but in every other way similar, phosphonate **3 a** in the site‐saturation libraries of both enzymes (Figures S11 C and S12 C). The fact that activities toward the similarly sized sulfate monoester **4** and phosphonate **3 a** are correlated in both enzymes (Figures S9 and S10), despite differences in reaction center and TS in solution, is also consistent with this observation. The only substrate for which phosphonate **3 c** shows significant correlation is phosphate diester **2 b** (only in *Rl*PMH, *r*=0.64; *p*=0.0025, Figure S12 B), the bulkiest substrate out of the four possibilities. This strong apparent dominance of size over other substrate/reaction properties cannot readily be explained in terms of steric clashes, at least not those with the residue at position 103: there is no significant correlation between the volume of the residue at position 103 in *Sp*AS1 and the activity towards any of the tested substrates (Figures 7 A and S5).

The lack of correlation between the activities towards the two phosphonates is most likely the result of beneficial hydrophobic interactions that are only present for the substrate with the large hydrophobic substituent (Figure S5 D). For position 107 in *Rl*PMH, however, the lack of correlation is due to the steric exclusion of the bulky phosphate **3 c**, which is absent in the case of phosphonate **3 a**. This is evident from comparison of the effect of residue volume on both activities across mutants among *Rl*PMH T107X: there is a significant inverse correlation for residue volume and activity towards phosphonate **3 c**, but none for phosphonate **3 a** (Figures 7 B and S6 C, D). Furthermore, the effects on **3 a** and **3 c** are not correlated at all in the two enzymes: that is, **3 a** and **4** (chemically different, same size) experience a greater mutational effect than **3 a** and **3 c** (chemically identical, different in size; see Figures S9 and S10). These observations suggest that any difference in solution TS, reaction center, or GS charge of the various substrates is surpassed by other GS (and TS) binding effects such as steric clashes and/or hydrophobic effects.

### Mutation of the active‐site nucleophile

The effect of multiple amino acid substitutions in the nucleophile‐flanking position 103/107 in an AS and a PMH on their catalytic specificity showed no consistent trends with regard to substrate or TS properties that might explain the effect of these mutations. We further tested whether or not the effect of an inactivating mutation to the nucleophile itself might be explained in terms of properties of substrate GS or TS. The active‐site functionality of five ASs and four PMHs^17^ was compromised by changing the active‐site nucleophile from a fGly residue (encoded in the form of a cysteine residue embedded in a recognition sequence) into a serine residue. Unlike cysteine, serine cannot be post‐translationally modified to fGly in *E. coli*.^68^, ^69^, ^70^ The mutation to serine was preferred over the more strongly deleterious mutation to alanine, in order to allow quantitative determination of the effect of the nucleophile mutation on specificity, because the serine variants exhibit significant residual activity that is still measurable for all promiscuous activities.

**Figure 8:**
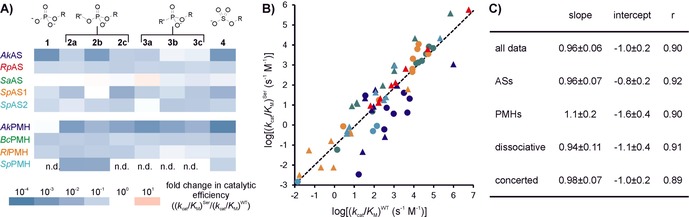
The effect of replacing the active‐site nucleophile fGly with serine for PMHs (circles) and ASs (triangles) was measured by determining Michaelis–Menten parameters with purified enzymes. A) Heat maps representing the changes in catalytic efficiency (*k*_cat_/*K*_M_) as a result of the Cys‐to‐Ser mutation in ASs and PMHs relative to the wild type for enzyme‐catalyzed hydrolysis of phospho‐ and sulfoesters **1**–**4**. See Tables S6–S14 for detailed kinetic data and experimental conditions. B) Correlation plot between the catalytic efficiencies of the wild‐type (fGly) and mutant (Ser) enzyme variants toward substrates **1**–**4**. *Ak*AS: ▴, *Rp*AS: ▴, *Sa*AS: ▴, *Sp*AS1: ▴), *Sp*AS2: ▴), *Ak*PMH: •, *Bc*PMH: •, *Rl*PMH: •, and *Sp*PMH: •. We observe a linear correlation between log (*k*_cat_/*K*_M_)^Ser^ for the serine mutants (*y*‐axis) and log (*k*_cat_/*K*_M_)^WT^ for the wild‐type enzyme with an fGly nucleophile (originating from cysteine) (*x*‐axis). The black dotted line represents a linear correlation for all data (slope=0.96±0.06; intercept=1.0±0.2; *r*=0.90, see also (C)). The linearity of this curve indicates that the mutation resulted in an ≈tenfold decrease in catalytic efficiency (*k*_cat_/*K*_M_) that is largely independent of the level of wild‐type activity, across *k*_cat_/*K*_M_ values ranging from 10^−2^ to 10^7^ s^−1^ m ^−1^. C) Slopes and intercepts for linear correlation graphs for the mutational effects classified either according to enzyme type (ASs vs. PMHs) or transition state type of the solution reaction catalyzed (dissociative vs. concerted). The corresponding fits are shown in Figure S13. No clear distinction could be observed between the mutational effect in PMHs and ASs. Similarly, no difference was observed between substrates that follow a dissociative or concerted transition state in solution. All fits showed *p*<10^−4^. Strong deviations from the correlation were associated with a particular enzyme rather than a particular substrate class. These deviations were non‐systematic (e.g., *Ak*AS: ▴ showed large deviations from the trend in both directions: 10^3^‐fold (phosphate **1**) and 1.5‐fold (phosphonate **3 b**) decrease vs. tenfold average decrease for the complete data set).

All nine mutant enzymes were overexpressed in *E. coli* and purified from cell lysate (essentially as described by van Loo et al.^17^), and kinetic parameters were determined for substrates **1**–**4**. For individual substrate/enzyme combinations, the effect of the mutation varied from a slight increase to a ≈10^3^–10^4^‐fold drop in catalytic efficiency relative to the wild type (Figure 8 A, Tables S6–S14). The magnitudes of the mutational effects vary considerably: even the activities toward similar substrates (i.e., phosphodiesters **2 a**–**c** and phosphonate monoesters **3 a**–**c**) are highly variable and do not show any obvious correlation with the size of the unreactive substituent (Figure 8 A). However, despite the large differences between “outliers”, the overall effect of mutating the nucleophile is well correlated across all enzyme/substrate combinations. The linear fit of a logarithmic correlation plot between catalytic efficiencies (*k*_cat_/*K*_M_) for mutant versus wild type for all eight substrates had a slope near unity (*r*=0.90, *p*<10^−4^, Figure 8 B). A *y*‐axis intercept of −1 indicates that replacing the fGly nucleophile with a serine residue results on average in an approximately tenfold decrease in catalytic efficiency, independent of the wild‐type level of activity (despite *k*_cat_/*K*_M_ values for the wild‐type enzymes ranging from 10^−2^ to 10^7^ s^−1^ m ^−1^). This correlation was virtually identical in ASs and PMHs (Figures 8 C and S13 A, B), thus suggesting that the effect of mutation of the nucleophile on any reaction catalyzed by one of these enzymes is not correlated with the apparent primary function.

As mentioned above, the ASs and PMHs catalyze reactions that proceed through different TSs in solution. Phosphate and sulfate monoester hydrolysis proceeds through loose or dissociative TSs,^38^, ^39^, ^40^ whereas phosphodiesters and phosphonate monoesters are hydrolyzed via concerted TSs in solution.^36^, ^37^ The contribution of the nucleophile to catalysis is expected to be more important for concerted reactions, in which case changing the nucleophile would affect their catalysis more strongly than for dissociative reactions. However, there was no significant difference in the decreases in catalytic efficiency as a result of changing the nucleophile for reactions that follow either more dissociative or more concerted reaction pathways (Figures 8 C and S13 C, D). This observation suggests that, for the given set of hydrolytic reactions, the nature of the TS of the catalyzed reaction in solution does not determine the effect of mutation on the enzymatic activity. This might be because TSs in the enzymatic reactions are more similar than in solution (as proposed, for example, by McWhirter et al.^71^). However, a considerable body of evidence suggests that such changes in the extent of transition state charge distribution and bond order are typically relatively small.^36^, ^37^, ^72^, ^73^ Other aspects of catalysis—substrate positioning for nucleophilic attack, for example—might be more important, overriding effects that the nature of the TS might have.

Even though the overall fit to the mutational effects is significant and reliable, there are considerable deviations from the fitted curve for individual enzymes/activities. If the nature of the substrate is the dominant determinant of the effect of mutation on a particular activity (and thereby responsible for its deviation from the fitted line), the patterns of effects for the nine different enzymes should be largely the same—that is, the activities with the weakest and strongest effects should be the same for all enzymes. However, when the same data shown in Figure 8 B are disaggregated in terms of enzyme and substrate (shown in Figure 8 A), the patterns of mutational effects (e.g., the reduction of the activity towards a given substrate in the Cys‐to‐Ser mutant in relation to the wild type) show large variation. Apparently, each enzyme responds differently to the same mutation and the effects of mutation on each activity are not the same, leading to unique patterns (Figure 8 A).

Taken together, these data suggest that, despite the conserved identity of the core active‐site residues (nucleophile, metal‐binding residues, leaving group activation; see van Loo et al.^17^) in the two enzyme classes, their context and surroundings have a substantial effect on how mutations affect their catalytic power.

### Context‐dependence of mutations

This comprehensive study of mutational effects in members of related groups of promiscuous enzymes suggests that the main factor shaping the local fitness landscape of active‐site residues is the overall structural context imposed by the enzyme, rather than the physicochemical properties of the substrate or the TSs of the catalyzed reactions.

The effect of mutating the nucleophile is similarly independent of enzyme class (AS or PMH), as indicated by the correlations between the corresponding catalytic efficiencies of wild type and the fGly‐to‐Ser mutants (Figure 8 C). Furthermore, the linear correlation with a slope near unity suggests that the mutational effect is independent of the wild‐type activity level. These two observations strongly imply that the effect of the mutation is not related to the identity of the primary activity of an enzyme. In addition, we cannot detect a significant difference in the slopes of the correlation lines between reactions that are believed to proceed via concerted or more dissociative‐like TSs in solution. This observation stands in contrast to the expectation that mutation of the nucleophile should affect the rate of a reaction significantly more if the reaction proceeds through a concerted TS, because there is more involvement of the nucleophile in the TS. Along with the unique enzyme‐dependent differences between the patterns of mutational effects across the panel of substrates studied, this unexpected result strongly suggests that the protein environment has a stronger influence on the effect of mutation than the nature of the TS of the catalyzed reaction. These findings are further supported by the comparative site‐saturation mutagenesis of the nucleophile‐flanking residue in one member of each of the AS and PMH subgroups. Neither the charge nor the nature of the TS observed for the solution reaction is a strong predictor of the effect of amino acid substitutions when comparing the effects of the various mutations in the nucleophile‐flanking residue on the substrate specificities of *Sp*AS1 and *Rl*PMH.

A similar lack of predictability of enzymatic activity based on the nature of the catalyzed reactions was described for glutathione *S*‐transferase (GST) by Kurtovic et al., who observed that clusters of functionally similar GST variants in a multidimensional (promiscuous) substrate–activity space still contained enzymes that catalyzed very different reactions as individual promiscuous activities, and that different reaction types did not significantly contribute to functional clustering of mutants.^66^ In another study, Zhang et al.^67^ observed correlations between mutational effects on mechanistically different reactions catalyzed by GST. This became apparent from examination of factor loadings for activities when projected onto “functional space” mapped by PCA, in which, for example, transacetylation and reduction reactions are similarly affected by mutagenesis. In that particular case, the chemical properties of catalyzed reactions did not serve as a reliable predictor of mutational effects in promiscuous enzymes.

What might explain the correlations that we and others report for activities in mutant populations of promiscuous enzymes? Active sites, in particular those of promiscuous enzymes, can accommodate substrates in multiple orientations with regard to the catalytic groups.^1^, ^74^, ^75^, ^76^ Subsets of active‐site residues might contribute to catalysis of multiple reactions to different extents.^41^, ^77^, ^78^, ^79^ The bound substrates are exposed to highly localized, intrinsically nonspecific medium effects, leading to a lack of a correlation with reaction type. Steric effects that would constrain the orientation of substrates relative to active‐site residues are another factor that can contribute to the “masking” of chemical properties of substrates—the data reported in Figure 7 show evidence that steric effects due to non‐reacting groups in substrates can cause a correlation of activity with the volume of substituted residues in one (*Rl*PMH), but not in another related enzyme (*Sp*AS1), suggesting different steric constraints in the two enzymes. A mutagenesis study on nucleotide pyrophosphatase/phosphodiesterase (NPP) by Wiersma‐Koch et al.^80^ showed that bulky, hydrophobic residues in the vicinity of the active site tune the specificity in wild‐type NPP toward a preference for phosphate diesters over phosphate monoester substrates by four orders of magnitude. This observation was ascribed to favorable interactions with the non‐reacting R group of the phosphate diester and to unfavorable interactions of the groups with the negatively charged nonbridging oxygen atom of the phosphate monoester. Similar steric or hydrophobic interactions would be sufficient to explain the different trends observed for the site‐saturation libraries *Sp*AS1 H103X and *Rl*PMH T107X.

## Conclusions

### A highly plastic “gatekeeper” residue allows rapid specificity switching from sulfatase to phosphotransferase

We have identified His103 in the sulfatase *Sp*AS1 as a highly plastic, specificity‐determining position at which virtually every mutation substantially improves both activity and specificity of the enzyme toward phosphate or phosphonate esters (vs. its original sulfate monoester substrate). Specificity changes of up to 10^5^‐fold are readily obtained by this “minimal evolutionary step”^47^ and suggest that considerable respecialization can be achieved through a single mutation. Up to 100‐fold enhancements of promiscuous activities are obtained in individual mutants, and in nine out of 19 substitutions in which His103 of the arylsulfatase *Sp*AS1 is replaced, promiscuous activities are improved more than tenfold. The profound effects of single‐residue substitutions demonstrate that promiscuous arylsulfatases are versatile evolutionary starting points, in which a single‐point mutation has an immediate, large positive effect on the “new” activity, avoiding loss of function in evolutionary intermediates. These insights chart a forward evolutionary link across an activity landscape for functional adaptation.

### Consideration of fitness landscapes provides a framework for assessing whether activity transitions are feasible

Despite the remarkable plasticity of amino acid position 103, not all substitutions in this residue that improve promiscuous activities in *Sp*AS1 are directly accessible through single‐nucleotide substitutions. Fitness landscapes (such as those shown in Figures 4 and S8) link function and connectivity of amino acids, illustrating which amino acids can be interconverted directly through single‐nucleotide mutations between any of their codons. This allows for “mapping” of the local fitness landscape of position 103, showing that the histidine residue in *Sp*AS1 WT represents a fitness “well” with respect to its promiscuous activities: all amino acids accessible from histidine codons give rise to improved promiscuous activities. However, for the native sulfatase activity, His103 represents a fitness maximum. This asymmetry in the mutational effects makes improvement of promiscuous activities for this position almost automatic. At the same time, survival of the gene directly after mutation will be dependent on partial relief from selection pressure for maintenance of sulfatase activity, through, for example, gene duplication prior to mutation of H103 in one of the two copies. The fitness landscape also shows that threonine (found in most active PMHs) is accessible by three continuously uphill pathways: that is, every mutational step results in improvement of the promiscuous phosphodiesterase activity relative to the enzyme variant that directly precedes it.

Principal component analysis (PCA) is validated as a tool for visualizing and analyzing specificity effects of structurally analogous mutations, as well as trade‐offs between different catalytic activities across a panel of mutants. PCA allowed the projection of the four‐dimensional activity space of the promiscuous enzymes studied onto a 2 D plane. In contrast with, for example, correlation dot‐plots between pairs of activities (six of which would be required per enzyme library studied), PCA condenses the information to a single plot. The unique pattern of locations in the new activity plane mapped by principal components allowed for ready comparison of the specificity effects of structurally analogous mutations in the *Sp*AS1 H103X and *Rl*PMH T107X libraries. Combination with a plot of the four activities as vectors into the same activity space allowed analysis of correlation patterns between activities, and facilitated detection of inconsistencies in correlation between activities. PCA is complemented by conventional analysis of correlation coefficients between activities. Both analyses reveal that the same, structurally analogous amino acid substitution can have substantially different specificity effects in two related enzymes, and that properties of substrate GS or TS do not consistently predict the effect of mutation.

Steric constraints and local structural environments of active sites appear to determine much of the variation in promiscuous enzyme specificity in response to mutation. Both the activity effects in the two site‐saturation libraries analyzed here, as well as those observed with nucleophile mutants (Cys/fGly→Ser) of a set of ASs and PMHs, reveal that properties of the substrates and catalyzed reactions fail to explain the effect of mutation on specificity. Structurally analogous mutations to the same residue have distinct effects on specificity, depending on the enzyme into which they are introduced. The fact that chemical properties of reactions and substrates are not always useful predictors of mutational effects suggests that subtle differences in the active‐site environments, leading to steric constraints, as well as multiple binding modes, might be very important in tuning the precise specificity of promiscuous enzymes.

Active‐site residues can contribute to catalysis in many ways, depending on their orientation with respect to the substrate. Promiscuous enzymes are opportunistic, or rather permissive, and this permissiveness can be further enhanced by conformational diversity,^81^, ^82^, ^83^ which has also been shown to contribute to physiological multifunctionality,^78^, ^79^ and can be altered as a consequence of evolution.^84^ If two substrates, despite chemical similarities, interact with an active site in different orientations—for example, as a consequence of subtle steric clashes—the effects of mutation on activities might indeed be very different.

### The degree of chemical differentiation between reactions in promiscuous enzymes might be limited

The nature of the TS and its charge distribution should be of paramount importance for the highly specific interactions between an enzyme and its substrate. However, if molecular recognition is tolerant rather than highly specific, seemingly straightforward extrapolations from the solution mechanism might be invalid. The observation that specificity effects of mutations are more dependent on the substrate structure than on the properties of the reactions further supports the notion of catalytic promiscuity as the result of inherent reactivity of the active site: promiscuous enzymes might not be able (or need) to specialize more in terms of chemistry, nor change the nature of transition states—leaving sterics as the paramount contributor to the observed specificity.^1^, ^9^, ^10^, ^43^, ^72^, ^77^ This suggests that the specificity pattern, or rather a basic catalytic capability, might indeed be intrinsic, and that promiscuity might exist, as stated by Copley,^85^ “simply because it is impossible to exclude all potential substrates”.

As a result of this apparently limited ability to differentiate reactions even in closely related enzymes, in any larger enzyme family, or even just a “quasi‐species” made up of functionally similar mutants,^57^, ^58^ the chances that an enzyme sequence that “fits the bill” to allow for a particular promiscuous activity will be present are substantial.

### A case for keeping template variability high during protein engineering

The fact that very different scenarios are reported in the literature with respect to the number of mutations required for functional transitions highlights the fact that generalizations answering the questions “how many mutations are required?” or “are mutations generally detrimental?” are unlikely to hold for the engineering of any one particular enzyme. They will have to be defined for each individual enzyme, because the shape of the local fitness landscape of a residue is determined by its structural environment and its quantitative contribution to TS stabilization. The observation of very different physicochemical solutions to the same problem (in this case, improvement of phosphodiesterase activity in the *Sp*AS1 H103X library) in combination with the context‐dependence of mutational effects observed in this work, and by other researchers,^22^, ^62^ demonstrates how important it is to take as many variants as possible forward into subsequent rounds of enzyme optimization: different solutions might improve a given activity initially, but the chosen starting point for subsequent engineering might substantially influence its long‐term outcome.

## Experimental Section

**Materials**: Phosphate monoester **1**, phosphonate monoester **3 c**, and sulfate monoester **4** were purchased from Sigma–Aldrich. Phosphate diester **2 b** was prepared as described previously.^86^ All other phosphoester substrates were prepared as described in van Loo et al. (2016).^17^ StrepTactin‐coated spin columns (IBA) and StrepTactin‐Superflow resin (IBA) for protein purification were purchased from Stratech Scientific. *Pfu* DNA polymerase was obtained from Agilent. DpnI was from ThermoFisher Scientific. Custom mutagenic oligonucleotides were obtained from Invitrogen.

**Mutant construction**: Mutants *Rl*PMH C57S and T107A were constructed previously.^11^ All other site‐directed mutants were created by using the QuikChange site‐directed mutagenesis protocol (Agilent), with the corresponding pASK‐IBA5plusPMH/AS wild‐type plasmids^17^ as templates and use of the primers listed in Table S15–S17. The presence of the mutations was confirmed by DNA sequencing (sequencing facility of the Department of Biochemistry, University of Cambridge).

**Protein production and purification**: Production of the Cys‐to‐Ser mutants of the various ASs and PMHs was achieved by growing *E. coli* TOP10 containing the appropriate pASK‐IBA5plus constructs in 2YT medium (750 mL) containing ampicillin (100 mg L^−1^) at 37 °C to an OD_600_ of ≈0.5, at which point the culture was cooled to 25 °C in ≈30 min. Expression of the corresponding AS/PMH Cys‐to‐Ser variants was achieved by addition of anhydrotetracycline (up to 200 μg L^−1^) followed by overnight growth at 25 °C. Harvesting of cells and subsequent protein purification were performed as described previously.^11^, ^14^, ^17^

Production of the other *Sp*AS1 and *Rl*PMH variants was done by inducing expression of the appropriate pASK‐IBA5plus constructs in *E. coli* Rosetta 2 co‐expressing the formylglycine‐generating enzyme (FGE) from *Mycobacterium tuberculosis* H37v (*Mtb*FGE)^87^ from the pRSFDuet*Mtb*FGE plasmid.^11^ Large‐scale protein production of *Sp*AS1 H103T and *Rl*PMH T107G was achieved by growing the appropriate variants in 2YT medium (750 mL) containing ampicillin (100 mg L^−1^) and kanamycin (50 mg L^−1^) at 37 °C to an OD_600_ of ≈0.5, at which point the culture was cooled to 25 °C in ≈30 min. Expression of *Mtb*FGE was induced by adding isopropyl β‐d‐1‐thiogalactopyranoside (IPTG, up to 1 mm), approximately 20 min prior to induction of expression of *Sp*AS1 H103T or *Rl*PMH T107G by addition of anhydrotetracycline (up to 200 μg L^−1^) followed by overnight growth at 25 °C. Harvesting of cells and subsequent protein purification was carried out as described previously.^11^, ^13^, ^17^

For the small‐scale activity tests, cells were typically grown in lysogeny broth (LB) medium (5 mL) containing ampicillin (100 mg L^−1^) and kanamycin (50 mg L^−1^) at 37 °C to an OD_600_ of ≈0.5, at which point the culture was cooled to 25 °C in ≈30 min. Expression of *Mtb*FGE was induced by addition of IPTG (up to 1 mm), approximately 20 min prior to induction of expression of the *Sp*AS1/*Rl*PMH variant by addition of anhydrotetracycline (up to 200 μg L^−1^) followed by overnight expression at 25 °C. Cells were harvested by centrifugation and resuspended in lysis solution (500 μL, BugBuster (Novagen, 0.5×), Lysonase (Novagen, 1 μL per mL lysis solution), cOmplete EDTA‐free protease inhibitor (Roche, 1 tablet per 15 mL lysis solution)) and incubated at 25 °C for 15 min. After addition of wash buffer (1 mL, Tris**⋅**HCl (100 mm), NaCl (150 mm), MnCl_2_ (100 μm), pH 8.0) and mixing, cell debris was removed by centrifugation at 4 °C. Recombinant *Strep*‐tagged *Sp*AS1 and *Rl*PMH variants were purified from cleared lysates over *Strep*‐Tactin spin columns by following the manufacturer's protocol (IBA, GmbH, www.iba‐lifesciences.com) with the wash buffer mentioned above and elution buffer (3×150 μL, Tris**⋅**HCl (100 mm), NaCl (500 mm), MnCl_2_ (100 μm), d‐biotin (2 mm), pH 7.5). The eluates showed excellent purity (>95 %) by SDS‐PAGE analysis (Coomassie staining), with only one visible minor additional gel band that was ascribed to degradation.

**Activity assays for small‐scale protein purifications**: Activities towards substrates **1**, **2 b**, **3 a**, **3 c**, and **4** were measured by monitoring 4‐nitrophenolate formation at 400 nm with a SpectraMax Plus microplate reader (Molecular Devices). The reactions were performed under subsaturating conditions: that is, substrate concentrations were at least two times lower than *K*_M_ of the wild type (see Table S2 for details). All reactions were carried out at 30 °C in Tris**⋅**HCl (pH 7.5, 100 mm)+NaCl (500 mm)+MnCl_2_ (100 μm). Initial rates (*v*_0_) were determined in triplicate from linear fits to early steady‐state phases of time courses and averaged. Where significant background rates were detected, a one‐tailed independent samples *t*‐test was applied with confidence level *p*=0.05, and activities for all mutants that were not found to be significantly above background were set to 0 in order to avoid artificial amplification of the catalytic performance of inactive mutants in the subsequent normalization step. Initial rates (vmutant0 ) were corrected on the basis of densitometric quantification of SDS‐PAGE gel bands from eluate fractions for the concentration of mutant protein, with use of the wild type as an internal standard: that is, the ratio between the intensity of the gel band of the mutant and that of the wild type was used to normalize mutant rates so they could be compared reliably (vmutant,corrected0 ). Resulting activities were divided by the initial rate of the corresponding wild‐type enzyme for the substrate in question to give the ratio vmutant,corrected0 */v* WT0 . Because these rates were measured under sub‐saturating conditions (at least two to three times lower than *K*_M_, Table S2), this ratio corresponds to the ratio of second‐order rate constants, kmutant2 */k* WT2 . This ratio reflects changes of activities relative to the wild type, and is a good approximation for changes in *k*_cat_ */K*_M_ (Figure S2**)**. If the *K*_M_ is decreased more strongly than ≈fivefold by mutation, the ratios reported will reflect *k*_cat_/K_M_ less well and instead represent more of *k*_cat_. Large drops in *K*_M_ with little change in *k*_cat_ would mean that the rate improvements reported here would be *smaller* than the real improvements of *k*_cat_ */K*_M_ and thus represent a *lower* limit. However, our statistical analysis safeguards against possible bias by individual outliers: if a drop in *K*_M_ should occur for some mutants, it will be balanced by the majority of data points.

**Principal component analysis and correlation analysis**: Principal component analysis (PCA)^63^ for dimensionality reduction and analysis of correlation of different substrates in response to mutation of H103 (in *Sp*AS1) and T107 (in *Rl*PMH) was carried out in MATLAB (MathWorks), with use of Equation 1 to calculate a maximum of *i*=4 (maximum number is equal to the number of original dimensions) principle components from the original dataset (Figure 2, Tables S3 and S4). The direct effect of a mutation is in effect a change in binding energy for the TS or GS, which can be defined as ΔΔ*G*=1.36×log [rate mutant/rate wild type], so a dataset of log [kmutant2 /kWT2 ] values is suitable for correct comparison of mutational effects.

PCi=a1×logkmut2kWT21+b1×logkmut2kWT22b+c1×logkmut2kWT23a+d1×logkmut2kWT24

The calculated PCs were ranked according to the degree to which they account for the observed variance, and the two highest‐ranking PCs (PC1 and PC2) were used to define the new, two‐dimensional activity space onto which the original, four‐dimensional activity space is projected. For both datasets, the first two principal components accounted for >80 % of the observed variance (96 % for *Sp*AS1 H103X, 86 % for *Rl*PMH T107X). Both PC1 and PC2 were normalized to their appropriate maximum scores. Scores of mutants in the new activity space, as well as the factor loadings (representing the original four dimensions (i.e., the activities)) were projected onto the new coordinate system. Conventional correlation analysis was carried out by testing the validity of a simple linear correlation between the effects of the various mutations on enzyme‐catalyzed hydrolysis of the various substrates (Figures S9 and S10).

**Enzyme assays for determination of kinetic parameters**: The rates of enzymatic hydrolysis of substrates **1**–**4** were determined by monitoring the increases in absorbance of the released 4‐nitrophenolate at 400 nm with the aid of a SpectraMax Plus microplate reader (Molecular Devices). All reactions were performed at 30 °C either in Bis‐Tris propane (20 mm), NaCl (100 mm) or in SID buffer (succinic acid (44 mm), imidazole (33 mm), diethanolamine (33 mm)). Further details on reaction conditions are listed in Tables S1 and S6–S14. The extinction coefficients of 4‐nitrophenol were determined for pH 6.0–8.0, increasing from 2000 to ≈19 000 m ^−1^ cm^−1^ with increasing pH. Initial rates (*v*_0_) were converted into observed rate constants (*k*_obs_ in s^−1^) by normalizing them for the enzyme concentration (*k*_obs_=*v*_0_/[enzyme]). The observed rate constants were plotted against the substrate concentration ([S]), and kinetic parameters *k*_cat_, *K*_M_, and/or *k*_cat_/*K*_M_ were obtained by fitting the increase in *k*_obs_ as a function of substrate concentrations ([S]) to Equations (2) or (3).

(2) $$k{}_{\mathrm{obs}}=\frac{k{}_{\mathrm{cat}}\times \left[S\right]}{K{}_{M}+\left[S\right]}$$

(3) $$k{}_{\mathrm{obs}}=\frac{k{}_{\mathrm{cat}}}{K{}_{M}}\times \left[S\right]$$

## Conflict of interest

*The authors declare no conflict of interest*.

## Supporting information

As a service to our authors and readers, this journal provides supporting information supplied by the authors. Such materials are peer reviewed and may be re‐organized for online delivery, but are not copy‐edited or typeset. Technical support issues arising from supporting information (other than missing files) should be addressed to the authors.

Supplementary
