# Supplementary material for: Specificity Effects of Amino Acid Substitutions in Promiscuous Hydrolases: Context‐Dependence of Catalytic Residue Contributions to Local Fitness Landscapes in Nearby Sequence Space
Source: Chembiochem. 2017 May 2;18(11):1001–15. doi: 10.1002/cbic.201600657 (PMC5488252; doi:10.1002/cbic.201600657)
Supplement: Supplementary file 1 — Supplementary [file CBIC-18-1001-s001.pdf]

CHEMBIOCHEM

Supporting Information

Specificity Effects of Amino Acid Substitutions in Promiscuous Hydrolases: Context-Dependence of Catalytic Residue Contributions to Local Fitness Landscapes in Nearby Sequence Space

Christopher D. Bayer+,[a, b] Bert van Loo+,[a, c] and Florian Hollfelder*[a]

cbic_201600657_sm_miscellaneous_information.pdf

Supporting information:

Table of Contents

Supplementary Figures **S3**

Figure S1. Small-scale affinity purification and activity determination of *Sp*AS1 H103X and *Rl*PMH T107X variants **S3**

Figure S2. Validation of the small-scale activity assay **S4**

Figure S3. Dependence of mutational effect in *Sp*AS1 H103 on the change in hydrophobicity of the amino-acid side chain for various substrates.. .... **S5**

Figure S4. Dependence of mutational effect in *Rl*PMH T107 on the change in hydrophobicity of the amino-acid side chain for various substrates.. .... **S6**

Figure S5. Dependence of mutational effect in *Sp*AS1 H103 on the change in volume of the amino-acid side chain for various substrates.. .... **S7**

Figure S6. Dependence of mutational effect in *Rl*PMH T107 on the change in volume of the amino acid side chain for various substrates.. .... **S8**

Figure S7. Correlation between amino acid side chain volume (residue volume) and hydrophobicity (logP).. .... **S9**

Figure S8. The local fitness landscape for position 107 of *Rl*PMH, for phospomonoesterase, phosphodiesterase and sulfatase activity **S10**

Figure S9. Correlations between substrates for *Sp*AS1 H103X. **S11**

Figure S10. Correlation between substrates for *Rl*PMH T107X. **S12**

Figure S11. Correlatoins between the mutational effects on enzymz-catalyzed hydrolysis of bulky phosphonate monoester **3c** and those on activity towards less bulky substrates for *Sp*AS1 position H103 .... **S13**

Figure S12. Correlations between the mutational effects on enzyme-catalyzed hydrolysis of bulky phosphonate monoester **3c** and those on activity toward less bulky substrates for *Rl*PMH position T107 .... **S14**

Figure S13. Mutations of the active site nucleophile have similar effects in PMHs and ASs.. .... **S15**

Supplementary Tables **S16**

Table S1. Kinetic parameters measured for mutants of *Rl*PMH and *Sp*AS1 .... **S16**

Table S2. Sub-saturating substrate concentrations used in small-scale activity measurements ... **S17**

Table S3. Activity ratios k_2^{mutant}/k_2^{WT} from activity assays of spin-column purified *Sp*AS1 H103X variants **S18**

Table S4. Activity ratios k_2^{mutant}/k_2^{WT} from activity assays of spin-column purified *Rl*PMH T107X variants **S19**

Table S5. Values for hydrophobicity and size for the various amino acid side chains **S20**

Table S6. Kinetic parameters for the various reactions catalyzed by *Ak*AS **S21**

Table S7. Kinetic parameters for the various reactions catalyzed by *Rp*AS **S22**

Table S8. Kinetic parameters for the various reactions catalyzed by *Sa*AS **S23**

Table S9. Kinetic parameters for the various reactions catalyzed by *Sp*AS1 .... **S24**

Table S10. Kinetic parameters for the various reactions catalyzed by *Sp*AS2 .... **S25**
Table S11. Kinetic parameters for the various reactions catalyzed by *Ak*PMH **S26**
Table S12. Kinetic parameters for the various reactions catalyzed by *Bc*PMH **S27**
Table S13. Kinetic parameters for the various reactions catalyzed by *Rl*PMH **S28**
Table S14. Kinetic parameters for the various reactions catalyzed by *Sp*PMH **S29**
Table S15. Primers used for mutagenesis of the active site nucleophile **S30**
Table S16. Primers used for site-saturating mutagenesis of H103 in *Sp*AS1 .... **S31**
Table S17. Primers used for site-saturating mutagenesis of T107 in *Rl*PMH **S32**

Supplementary References **S33**

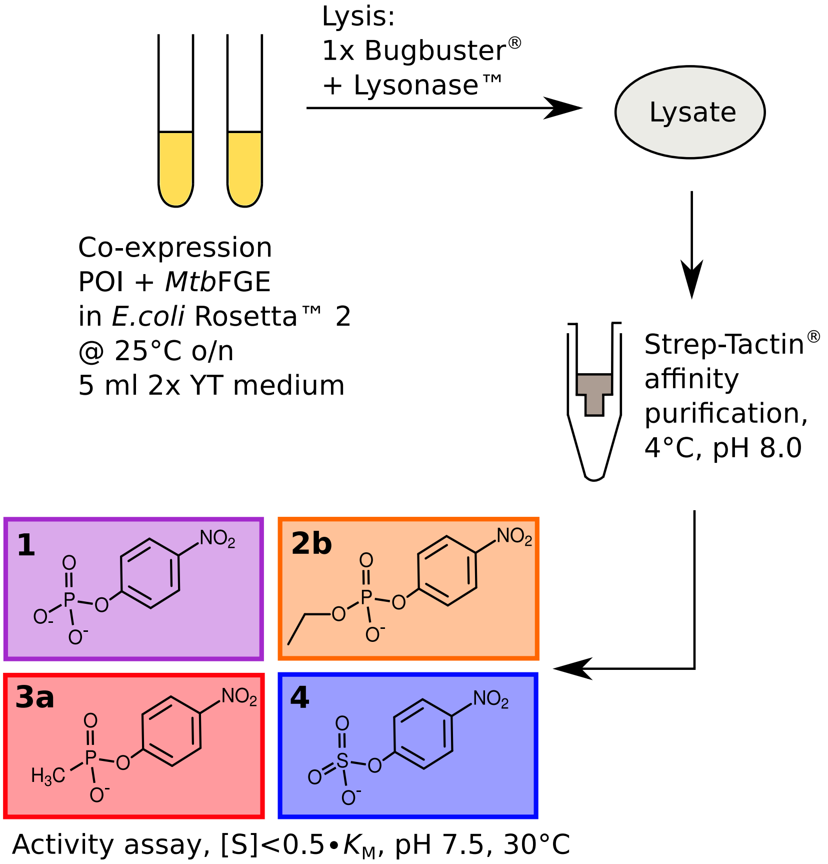

Figure S1. Small-scale affinity purification and activity determination of *Sp*AS1 H103X and *Rl*PMH T107X variants. The various mutations were introduced using site-directed mutagenesis and the respective Strep®-tagged enzyme variants (POI = proteins of interest) were overexpressed in *E. coli* expressing the formyl glycine (fGly) generating enzyme (FGE) from *Mycobacterium tuberculosis* (*Mtb*FGE) by overnight growth of 5 mL culture medium under inducing conditions (1 mM IPTG (*Mtb*FGE); 200 μg L^{-1} anhydrotetracycline (strep-tagged AS/PMH variant)) at 25 °C. The recombinant enzymes were purified from cleared cell lysate using Strep-Tactin® spin columns (IBA). Rates were corrected for the concentration of mutant protein relative to wild type, based on the densitometric quantification of the protein bands from SDS-PAGE. Initial rates were measured under subsaturating conditions (i.e. at least 2-fold lower than the K_M of the wild type, depending on the ability to reliably detect activity for different mutants, see Table S2 for actual concentrations). The ratio of initial rates of mutant to wilt type (V_0^{mutant}/V_0^{WT}) corrected for differences in protein concentration corresponds to the ratio of second order rate constants (k_2^{mutant}/k_2^{WT}), which reflect changes in k_{cat}/K_M upon mutation (see Figure S2).

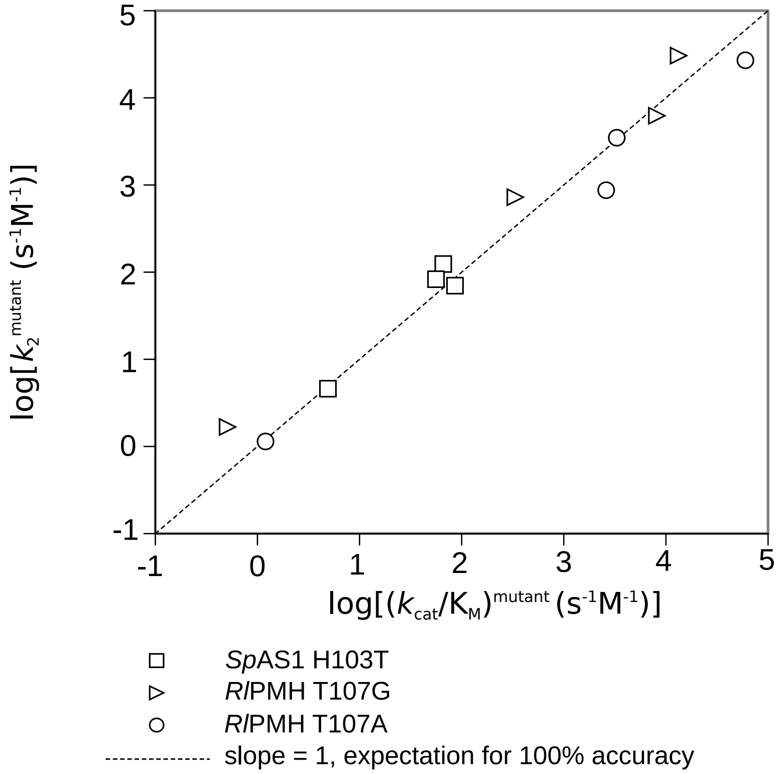

Figure S2. Validation of the small-scale activity assay. Correlation between log[k_2^{mutant} ($s^{-1}M^{-1}$)] obtained from small scale activity tests and the actual log[(k_{cat}/K_M)^{mutant}) ($s^{-1}M^{-1}$)] of the respective mutant towards the same substrate. k_2^{mutant} is calculated by multiplying the activity ratios k_2^{mutant}/k_2^{WT} determined in the small-scale activity tests (see Experimental section and Figure S1 for details) with k_{cat}/K_M-values of the respective wild-type enzyme (see Tables S9 and S13 for kinetic data of *Sp*AS1 WT and *Rl*PMH WT respectively). Values for (k_{cat}/K_M)^{mutant} ($s^{-1}M^{-1}$) of purified *Sp*AS1 H103T and *Rl*PMH T107G were determined as described in the experimental section. The data for *Rl*PMH T107A were determined previously (see Table S1 for details).[1] The data match a simulated line with a slope of 1 that is expected for a perfect correlation. This strongly suggests that the second order rate constants k_2^{mutant} measured at a single sub-saturating substrate concentration are a good approximation for (k_{cat}/K_M)^{mutant} as determined from a Michaelis-Menten fitted dataset.

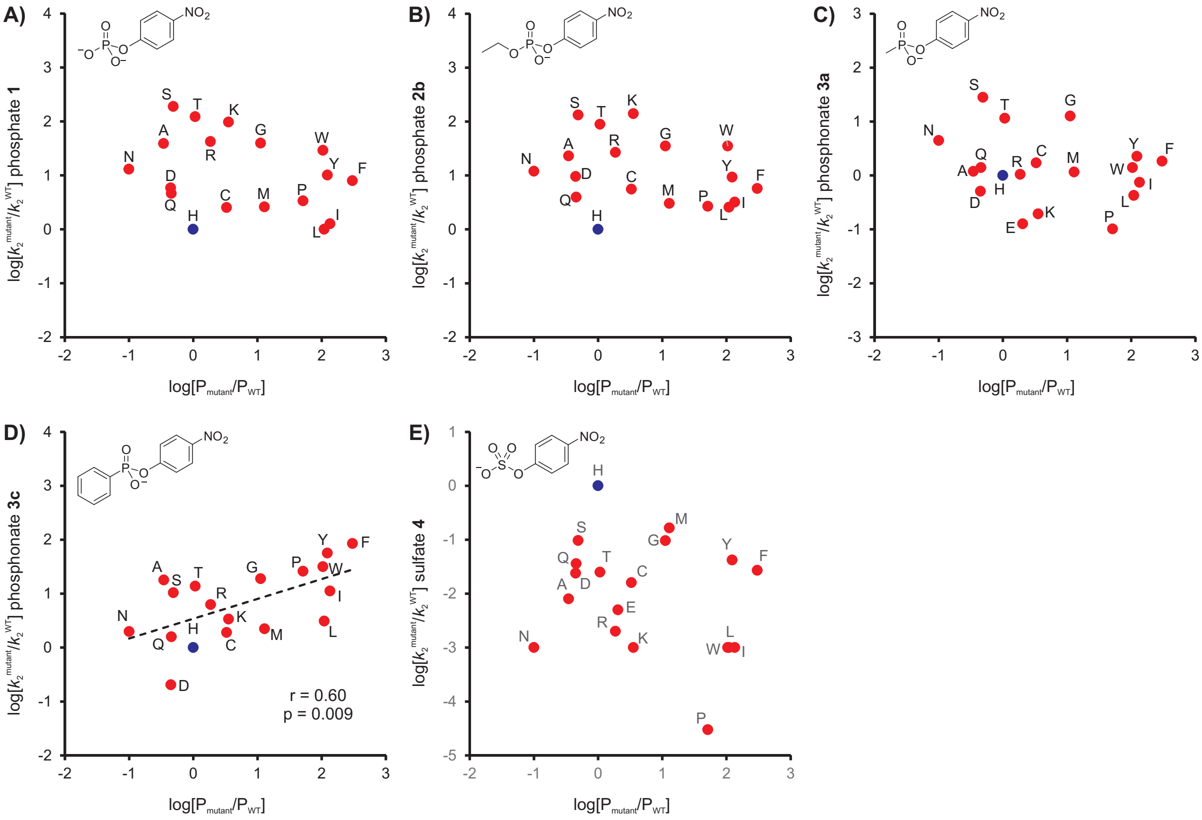

Figure S3. Dependence of mutational effect in *Sp*AS1 H103 on the change in hydrophobicity of the amino-acid side chain for various substrates. The change in hydrophobicity ($\log[P_{mutant}/P_{WT}]$) was derived from the individual logP values for each amino acid side chain (as calculated using ChemBioDraw Ultra 14.0, see Table S5 for actual values). **A**) phosphate monoester **1**, **B**) phosphodiester **2b**, **C**) phosphonate monoester **3a**, **D**) phosphonate monoester **3c**, **E**) sulfate monoester **4**. The mutational effects on all activities except phosphonate monoester **3c** show no correlation with the change in amino acid side chain hydrophobicity. Activity toward phosphonate monoester **3c** appears to benefit from increased active-site hydrophobicity, suggesting a role for favourable hydrophobic interactions during substrate binding and/or TS stabilization in *Sp*AS1.

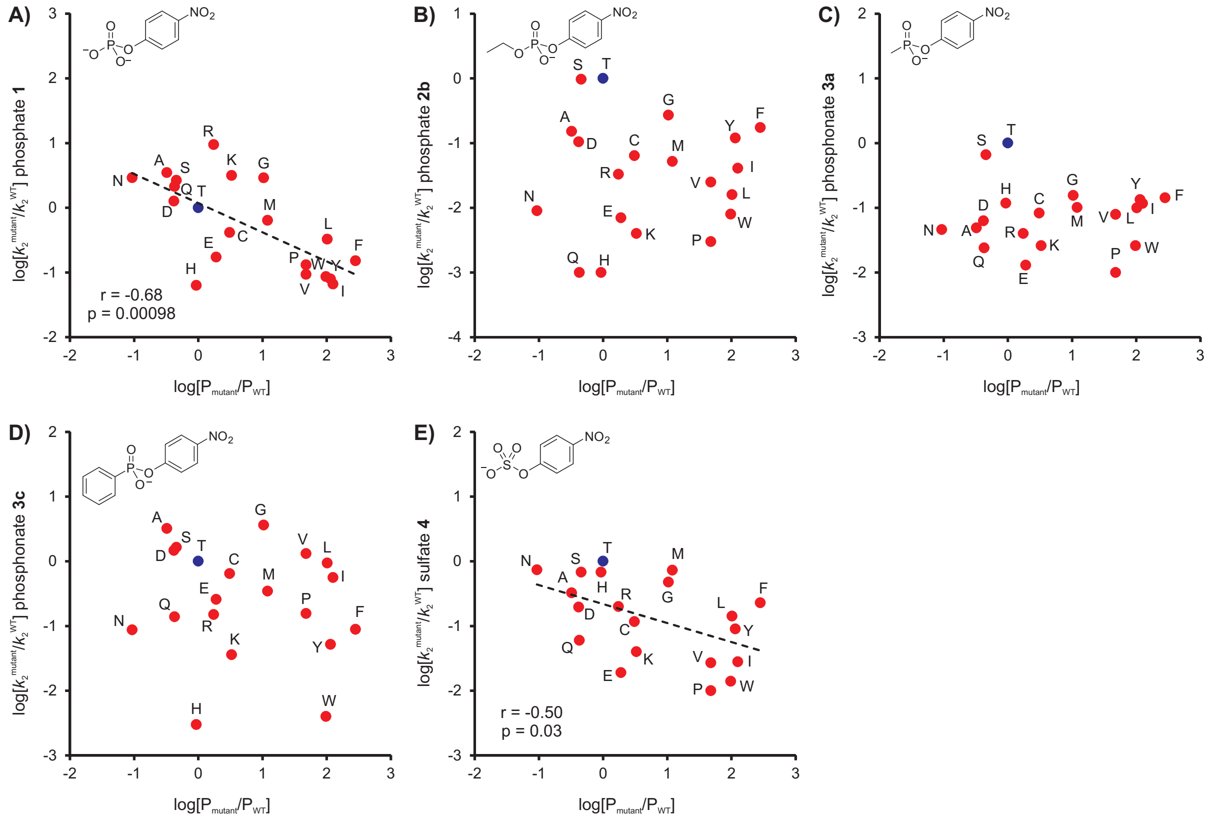

Figure S4. Dependence of mutational effect in *R/*PMH T107 on the change in hydrophobicity of the amino-acid side chain for various substrates. The change in hydrophobicity ($\log[P_{mutant}/P_{WT}]$) was derived from the individual logP values for each amino acid side chain (as calculated using ChemBioDraw Ultra 14.0, see Table S5 for actual values). **A**) phosphate monoester **1**, **B**) phosphodiester **2b**, **C**) phosphonate monoester **3a**, **D**) phosphonate monoester **3c**, **E**) sulfate monoester **4**. The mutational effects for phosphodiester **2b** and phosphonate monoesters **3a** and **3c** suggest there is no significant beneficial effect of hydrophobic interactions in substrate or TS binding. The negative correlation coefficients for phosphate monoester **1** and sulfate monoester **4** suggest that in *R/*PMH an increase in amino acid side-chain hydrophobicity of the nucleophile-flanking residue is detrimental to these activities.

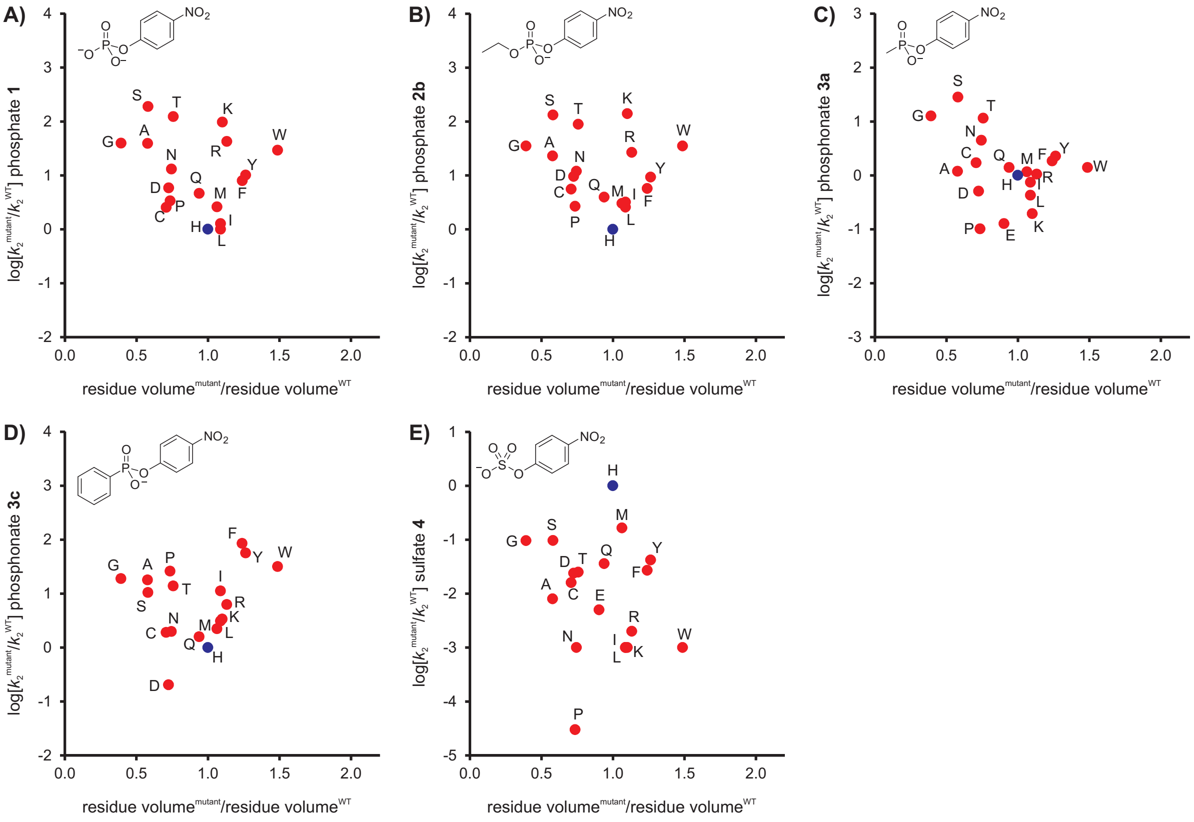

Figure S5. Dependence of mutational effect in *Sp*AS1 H103 on the change in volume of the amino-acid side chain for various substrates. The change in volume relative to the wild-type residue (residue volume^{mutant}/residue volume^{WT}) was calculated from published amino-acid side chain volumes[4] (See Table S5 for actual values). **A**) phosphate monoester **1**, **B**) phosphodiester **2b**, **C**) phosphonate monoester **3a**, **D**) phosphonate monoester **3c**, **E**) sulfate monoester **4**. The mutational effects of none of the activities show correlation with the change in amino acid side chain volume.

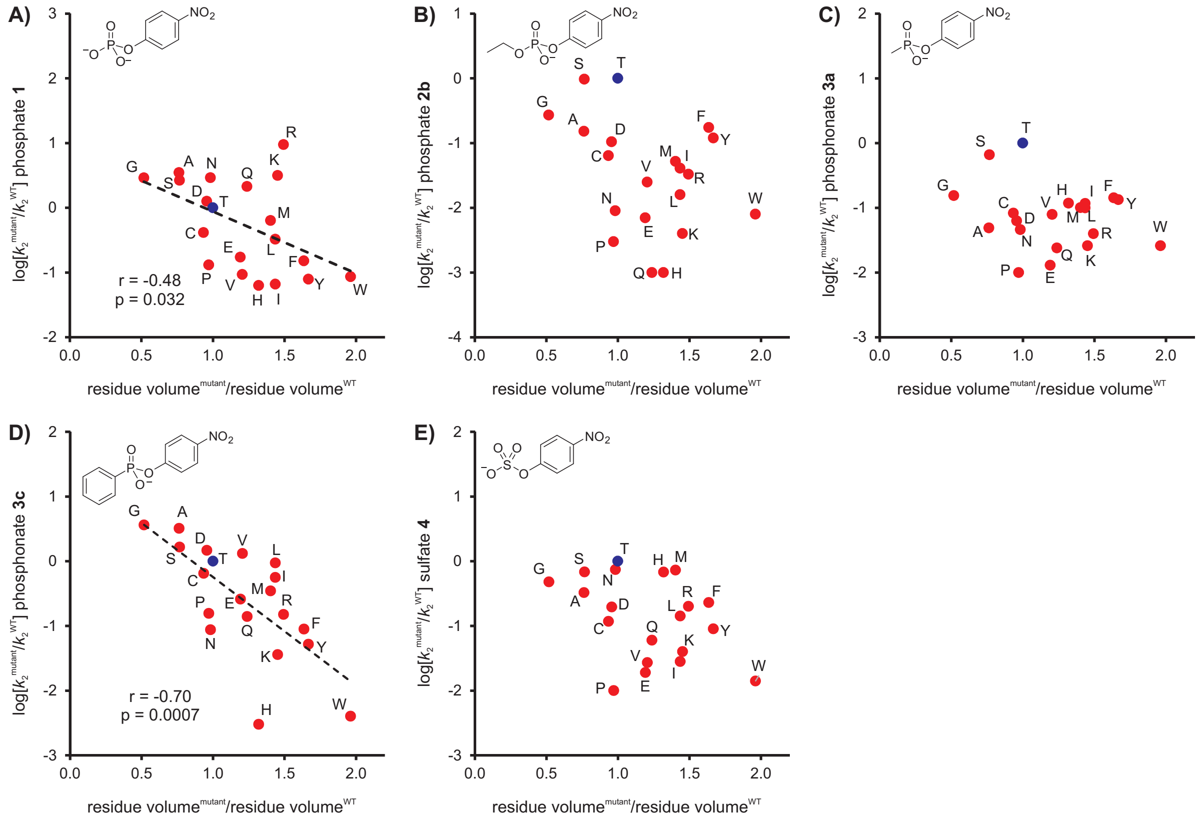

Figure S6. Dependence of mutational effect in *R/*PMH T107 on the change in volume of the amino acid side chain for various substrates. The change in volume relative to wild type (residue volume^{mutant}/residue volume^{WT}) was calculated from published amino-acid side chain volumes[4] (See Table S5 for actual values). **A**) phosphate monoester **1**, **B**) phosphodiester **2b**, **C**) phosphonate monoester **3a**, **D**) phosphonate monoester **3c**, **E**) sulfate monoester **4**. The mutational effects on enzyme-catalyzed hydrolysis of phosphate diester **2b**, phosphonate monoester **3a** and sulfate monoester **4** show no correlation with the change in amino aicd side chain volume. The observed negative correlation for phosphate monoester **1** is most likely due to the fact that amino acid side chain volume and hydrophobicity (represented as logP) are positively correlated (Figure S7), and the observed weakly significant correlation observed here (0.05 > p > 0.01) is most likely due to the negative correlation of mutational effects with the change in hydrophobicity (Figure S4A). The negative correlation shown for phosphonate monoester **3c** suggests that the nucleophile-flanking residue does not interact directly with the bulky phenyl group, but rather occupies the space where it would bind.

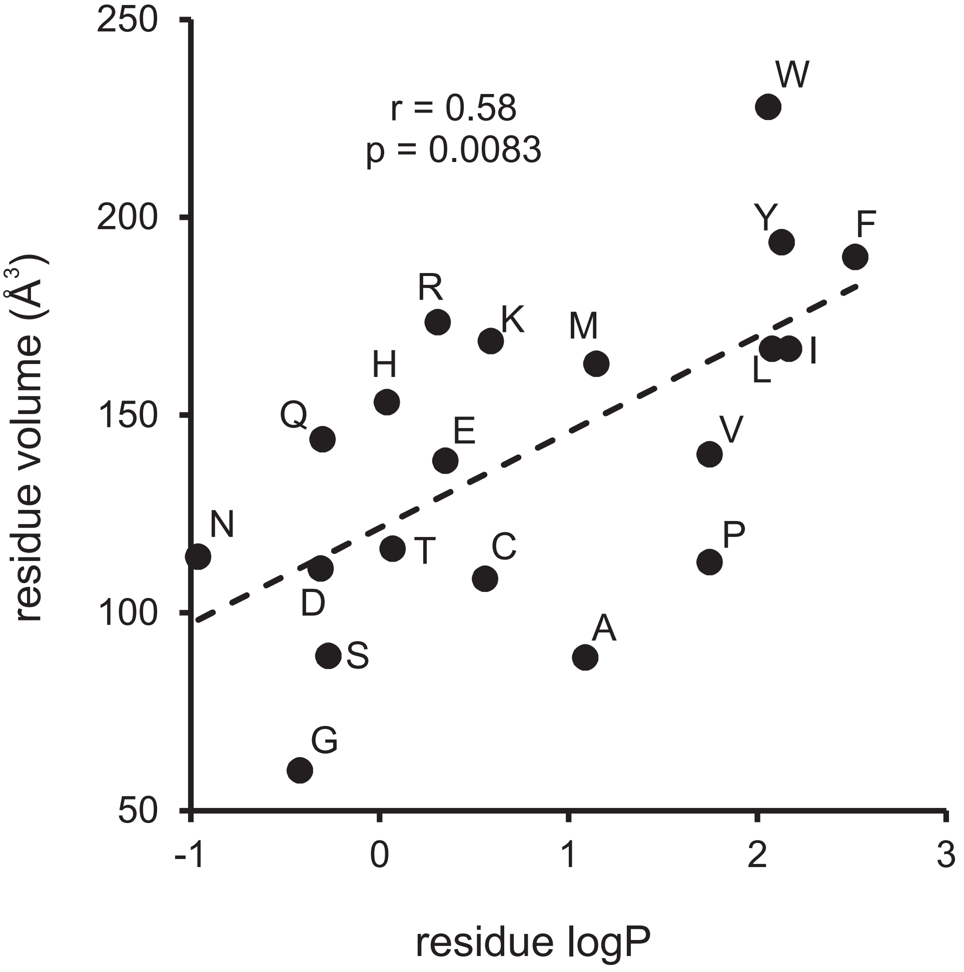

Figure S7. Correlation between amino acid side chain volume (residue volume) and hydrophobicity (LogP) (see Table S5 for the actual values).

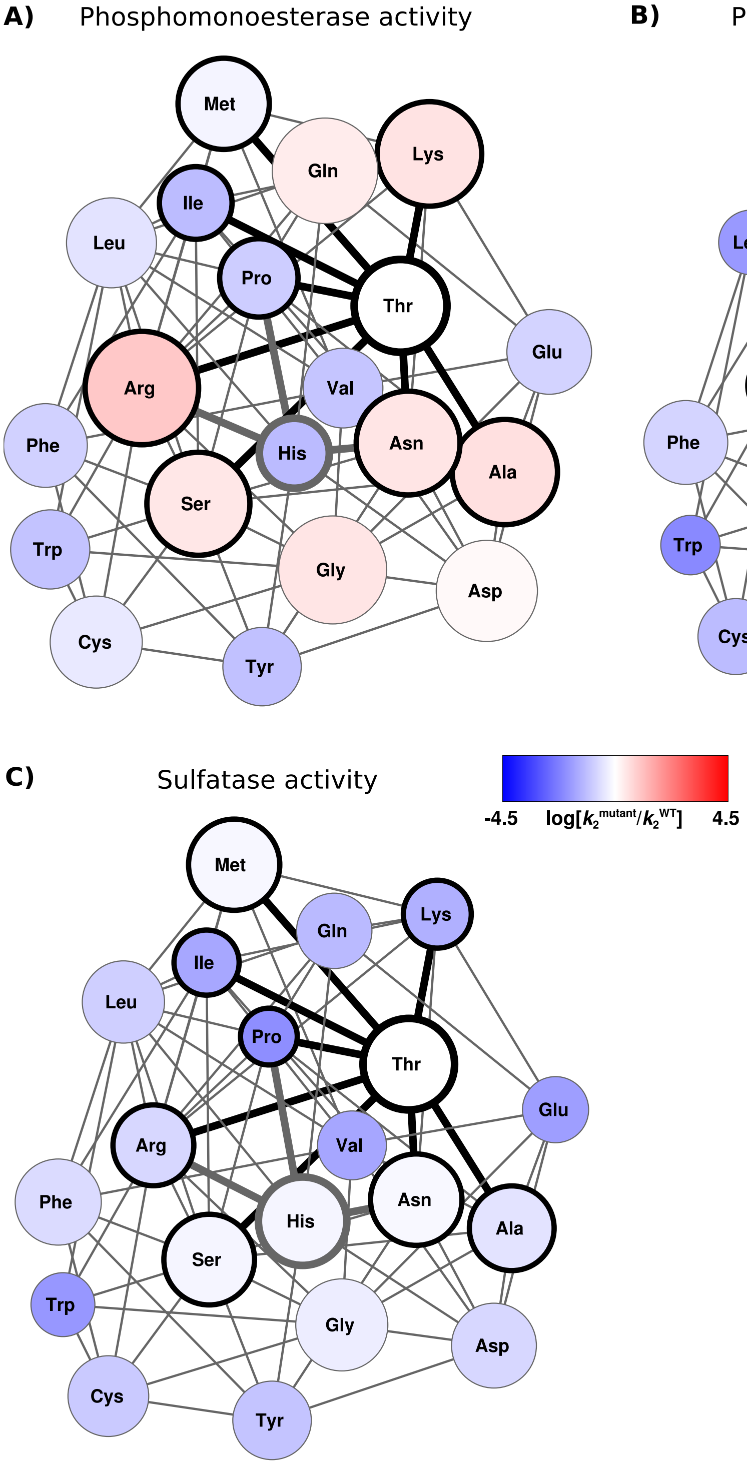

Figure S8. The local fitness landscape for position 107 of *Rl*PMH, for phosphomonoesterase (**A**), phosphodiesterase (**B**) and sulfatase activity (**C**). Shown is a network representation where nodes (circles) represent the amino acids at position 107 in a given variant. The color code (see legend) shows the activity of a given variant relative to *Rl*PMH WT as the log[k_2^{mutant}/k_2^{WT}] (see also Figure 2B and Table S4). Node diameter is also scaled according to log[k_2^{mutant}/k_2^{WT}], i.e. nodes larger than His (wild type) denote more active mutants, nodes smaller than His denote less active mutants. Edges (or connections between nodes) indicates that the two connected amino acids can be interconverted by a single nucleotide substitution. Highlighted in bold black are the wild type (Thr at position 107) and all amino acids directly accessible from any of the threonine-codons. Highlighted in bold gray are histidine, the amino acid of *Sp*AS1 WT at position 103 (the "target" in a hypothetical evolutionary trajectory).Thus,

the black/gray sub-network shows hypothetical trajectories of interconversion between threonine and histidine at position 107 of *Rl*PMH.

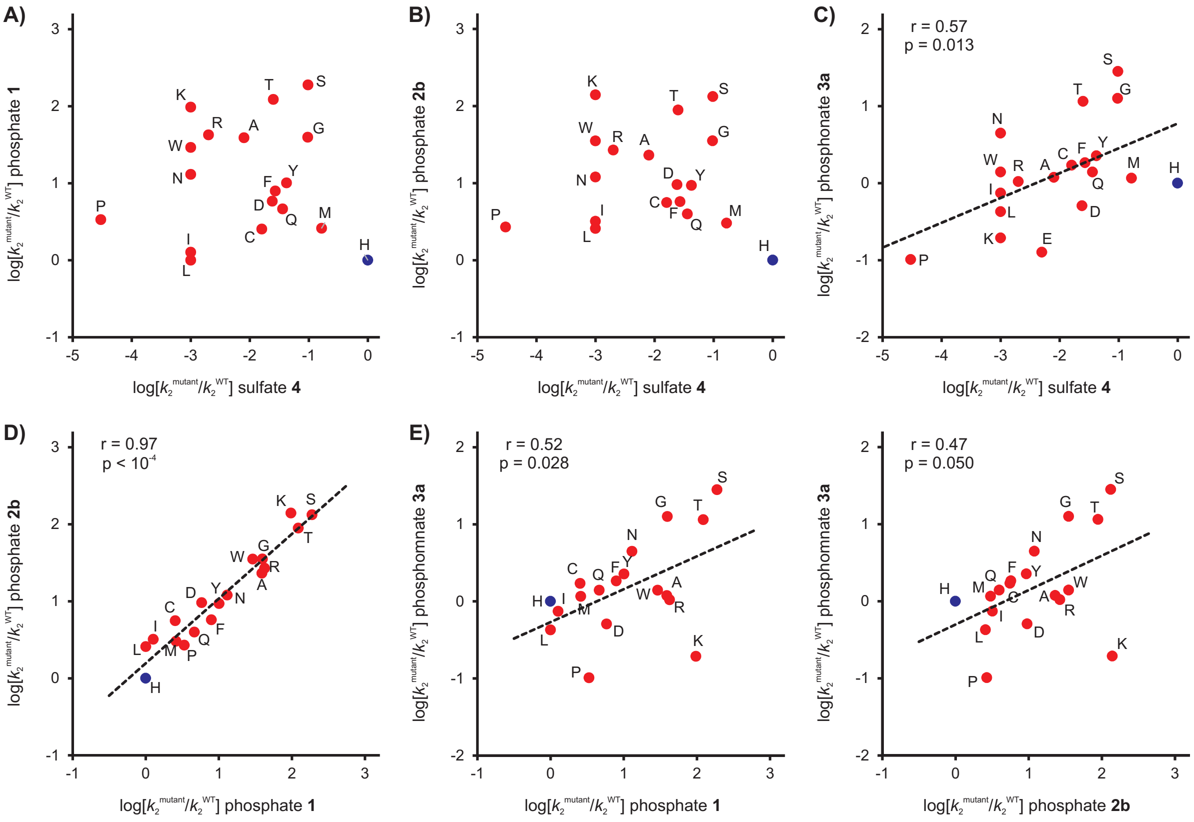

Figure S9: Correlations between the effects of the various mutations on the activity toward the various substrates for *Sp*AS1 H103X. Correlation line and fitting statistic are only shown for significant fits ($p \leq 0.05$). The correlation coefficients r and significance level p are also indicated in Figure 6C.

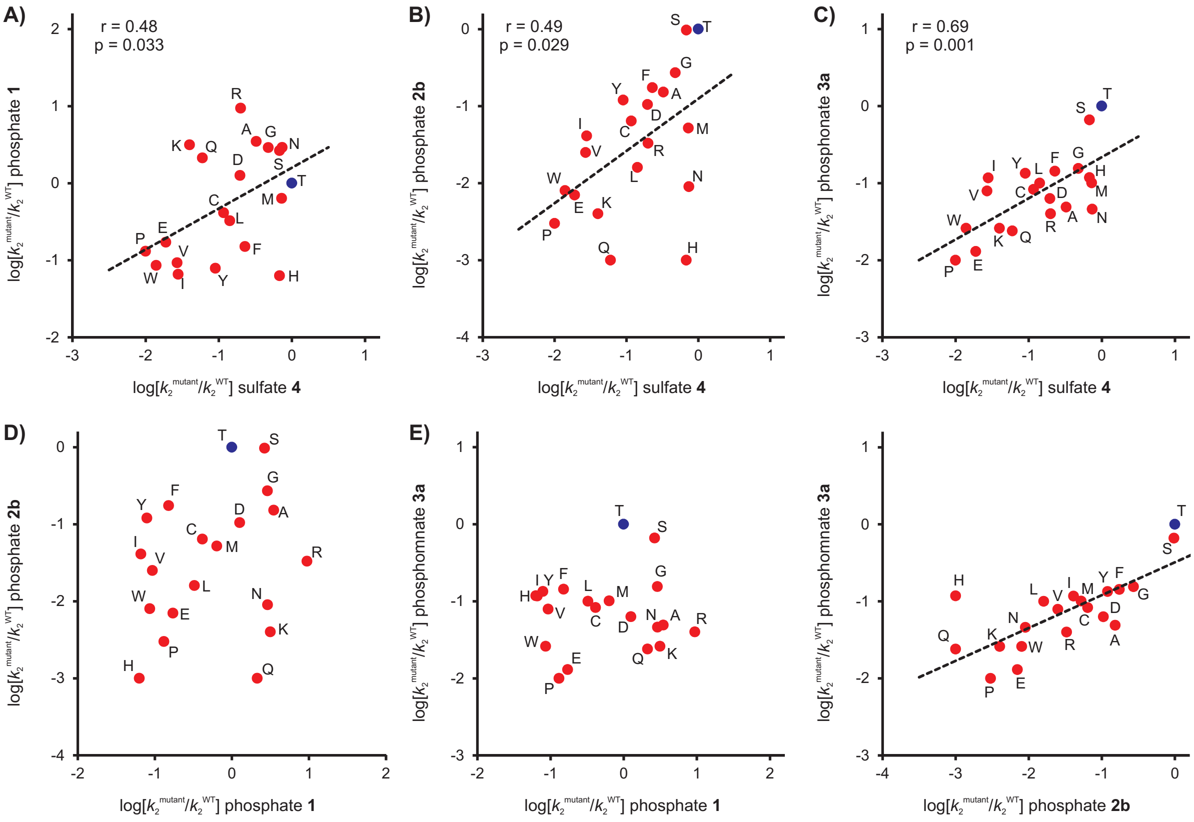

Figure S10: Correlations between the effects of the various mutations on the activity toward the various substrates for *Rl*PMH T107X. Correlation line and fitting statistic are only shown for significant fits ($p \leq 0.05$). The correlation coefficients r and significance level p are also indicated in Figure 6D.

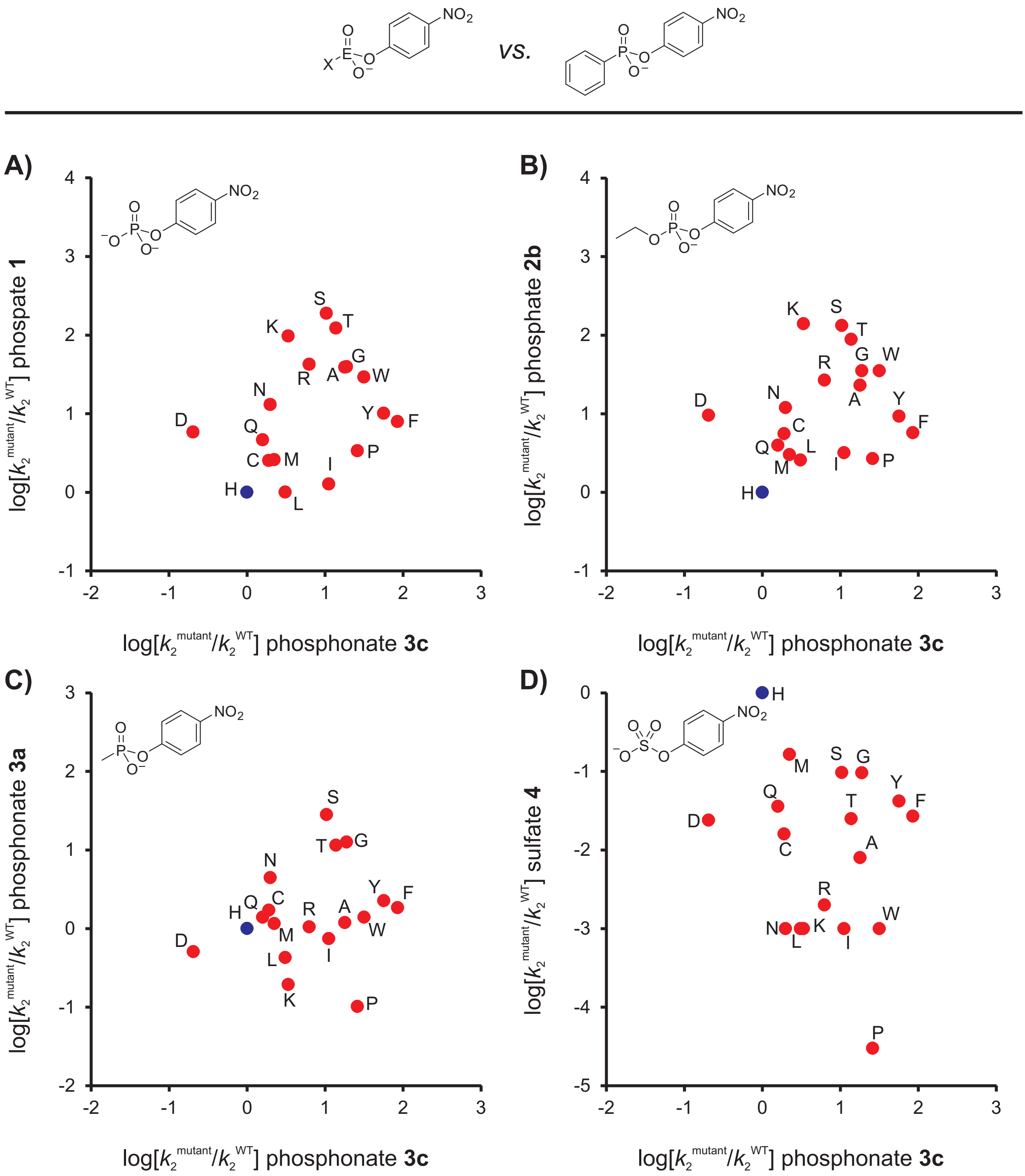

Figure S11. Correlations between the mutational effects on enzyme-catalyzed hydrolysis of bulky phosphonate monoester **3c** and those on activity toward less bulky substrates phosphate monoester **1** (**A**), phosphodiester **2b** (**B**), phosphonate monoester **3a** (**C**) and sulfate monoester **4** (**D**) for *Sp*AS1 H103X. No significant correlations are observed suggesting that the requirements for efficient turnover of phosphonate monoester **3c** and all other substrates are not identical.

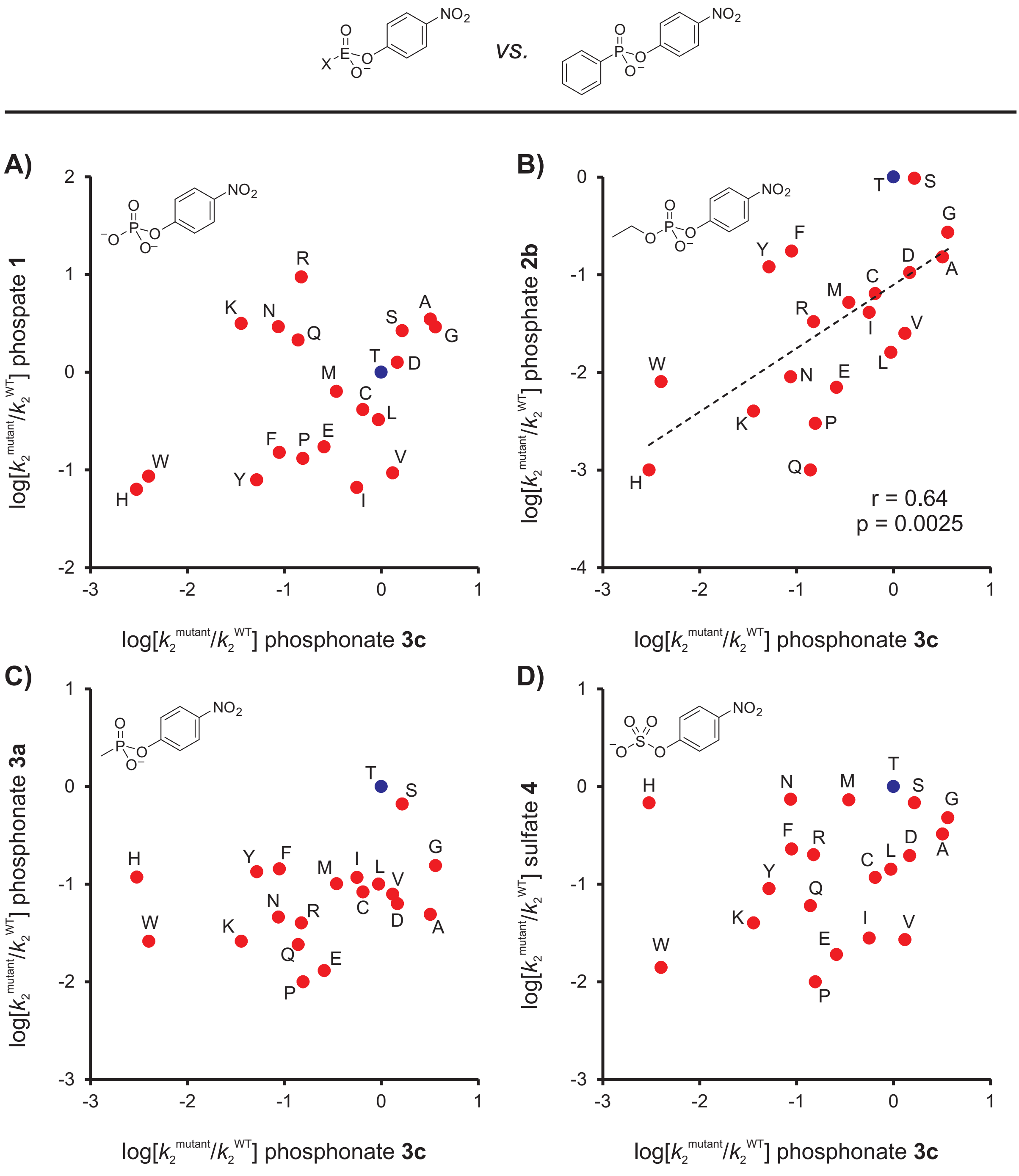

Figure S12. Correlations between the mutational effects on enzyme-catalyzed hydrolysis of bulky phosphonate monoester **3c** and those on activity toward less bulky substrates phosphate monoester **1** (**A**), phosphodiester **2b** (**B**), phosphonate monoester **3a** (**C**) and sulfate monoester **4** (**D**) for *R*/PMH T107X. Only for (**B**) a weak, yet significant correlation ($p < 0.05$) is observed (with aromatic residues being outliers), suggesting that the catalytic demands of **3c** and **2b** are similar (and, in conjunction with Figure 7, can be ascribed to similar spatial demands). Surprisingly the correlation of identical reaction chemistries (phosphonate **3a** *vs.* phosphonate **3c**) does not yield a correlation, suggesting that the bulk of the substrate dominates over identical chemical demands.

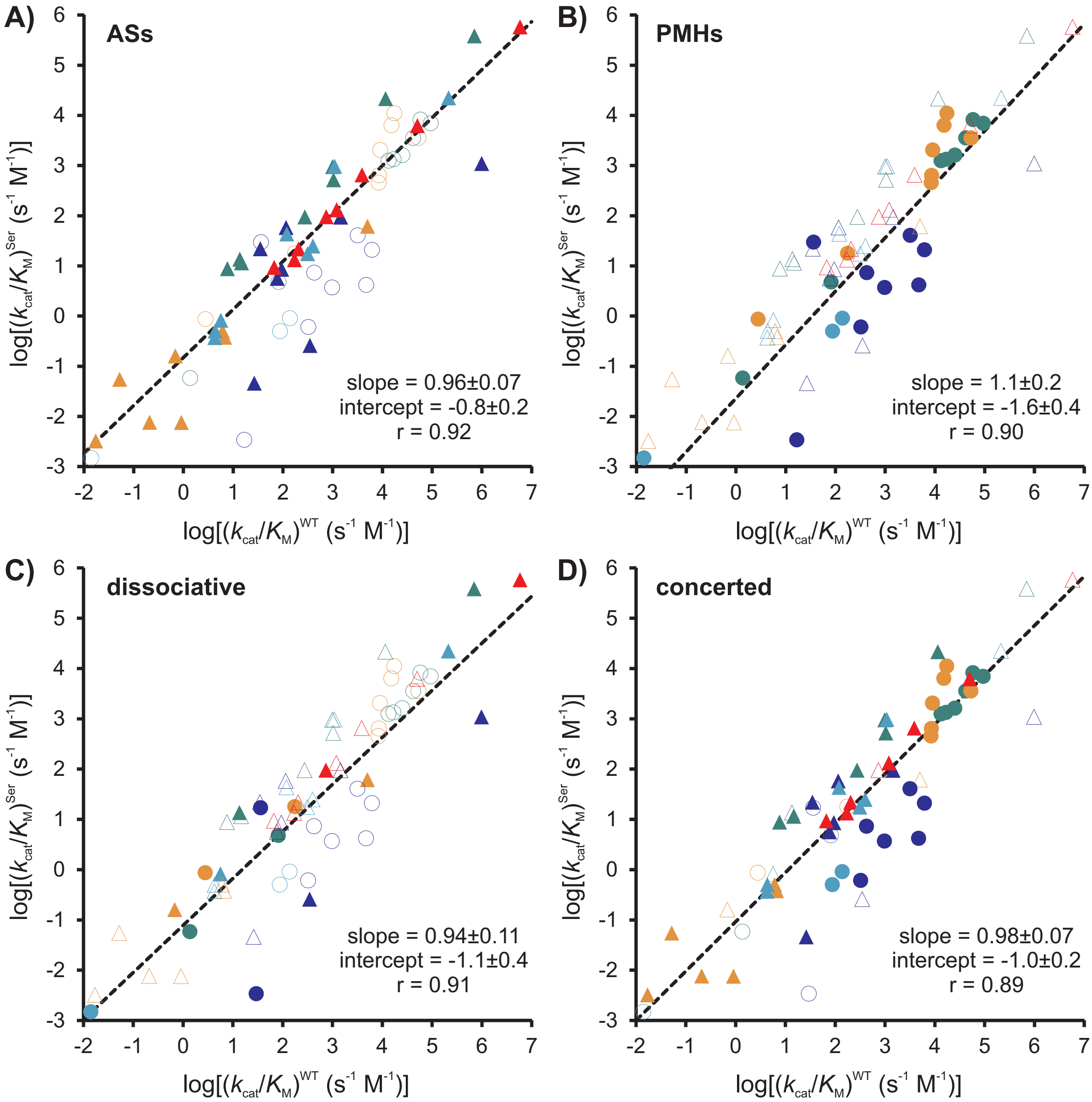

Figure S13. Effect of replacing the fGly-nucleophile with a serine residue in ASs (triangles) and PMHs (circles), classified by enzyme class (AS *vs.* PMH, panel **A** and **B**) and solution transition state of the catalyzed reaction (dissociative *vs.* concerted, panel **C** and **D**). For each panel only the solid data points were included in the fit indicated by the dashed line (open data points were left out). The data were fitted to y = ax + b with slope a and intercept b, with correlation coefficient r as indicated in each panel and $p < 10^{-4}$. A slope of 1 indicates that the effect of the mutation is similar in all enzymes. The fitting data (slope, intercept and correlation coefficient) are indicated in Figure 8C. Color coding legend: *Ak*AS (blue triangles), *Rp*AS (red triangles), *Sa*AS (green triangles), *Sp*AS1 (orange triangles), *Sp*AS2 (turquoise triangles), *Ak*PMH (blue circles), *Bc*PMH (green circles), *Rl*PMH (orange circles), and *Sp*PMH (turquoise circles).

Table S1. Michaelis-Menten parameters measured for *Rl*PMH and *Sp*AS1 mutants used for validation of the data from spin-column purified enzymes, Conditions: T=30 °C, SID buffer, pH 7.5, 100 μM $MnCl_2$ for *Rl*PMH, 200 μM $MnCl_2$ for *Sp*AS1.

Enzyme	Substrate	k_{cat} (s^{-1})	K_M (M)	k_{cat}/K_M (s^{-1} M^{-1})
*Sp*AS1 H103T	**1**	(4.1±0.2) × 10^{-1}	(7±1) × 10^{-3}	(5.7±1.0) × 10^{1}
	**2b**	(2.1±0.2) × 10^{-1}	(3.5±0.6) × 10^{-2}	6±1
	**3a**	(2.3±0.1) × 10^{-1}	(2.7±0.3) × 10^{-3}	(8.6±1.0) × 10^{1}
	**4**	2.6±0.3	(3.1±0.6) × 10^{-2}	(8.4±1.9) × 10^{1}
*Rl*PMH T107G	**1**	(1.56±0.04) × 10^{-1}	(4.6±0.4) × 10^{-4}	(3.4±0.3) × 10^{2}
	**2b**	(5.9±0.2) × 10^{0}	(7.2±0.7) × 10^{-4}	(8.2±0.9) × 10^{3}
	**3c**	7.7±0.4	(5.8±1.0) × 10^{-4}	(1.3±0.2) × 10^{4}
	**4**	(5.8±1.1) × 10^{-2}	(1.1±0.4) × 10^{-1}	(5.1±2.3) × 10^{-1}
*Rl*PMH T107A	**1**[a]	(4.1±0.1) × 10^{-1}	(1.6±0.2) × 10^{-4}	(2.6±0.3) × 10^{3}
	**2b**[b]	2.7±0.4	(8.2±1.9) × 10^{-4}	(3.3±0.9) × 10^{3}
	**3c**[b]	5.7±0.1	(9.5±0.7) × 10^{-5}	(6.0±0.5) × 10^{4}
	**4**[a]	>1 × 10^{-1}	> 1 × 10^{-1}	1.2

[a]Data from Jonas 2009[2]
[b]Data from Jonas et al. 2008[1]

Table S2. Sub-saturating substrate concentrations [S] used in small-scale activity measurements, performed at least 2-fold below the respective K_M. K_M-values were taken from van Loo et al.,[3] (See also Table S9 (*Sp*AS1) and Table S13 (*Rl*PMH)). At these substrate concentrations the measured initial rate V_0 is proportional to k_{cat}/K_M according to $V_0 = k_{cat}/K_M \times$ [Enzyme] × [S]).

Substrate	*Sp*AS1		*Rl*PMH	
	K_M^{WT} (mM)	[S] (mM)	K_M^{WT} (mM)	[S] (mM)
**1**	31	3	0.52	0.25
**2b**	121	3	1.5	0.5
**3a**	84	5	13	0.5
**3c**	>65	30	1.2	0.5
**4**	4	0.5	>80	20

Table S3. Activity ratios k_2^{mutant}/k_2^{WT} from activity assays of spin-column purified *Sp*AS1 H103X variants, after correction for protein concentration (see experimental section for details) and normalization to the respective wild-type enzymes. All reactions were carried out in triplicate at 30°C in 100 mM Tris-HCl, pH 7.5 + 500 mM NaCl + 100 µM $MnCl_2$. Substrate concentrations as indicated in Table S2. Errors correspond to the standard deviation from triplicate measurements.

**Amino acid X**	**Substrate**				
	**1**	**2b**	**3a**	**3c**	**4**
G	(4.0±0.8) × 10	(3.5±0.8) × 10	(1.3±0.3) × 10	(1.9±0.7) × 10	(9.6±1.1) × 10^{-2}
A	(3.9±0.9) × 10	(2.3±0.8) × 10	1.2±0.2	(1.8±0.6) × 10	(8±2) × 10^{-3}
V	n.e.	n.e.	n.e.	n.e.	n.e.
L	1.0±0.7	2.6±0.7	(4.3±0.3) × 10^{-1}	3.1±0.5	
I	1.3±0.1	3.2±0.4	(7.4±1.6) × 10^{-1}	(1.1±0.2) × 10^{1}	
C	2.5±0.3	5.6±0.7	1.7±0.3	1.9±0.2	(1.6±0.2) × 10^{-2}
M	2.6±1.0	3.0±0.5	1.2±0.2	2.2±0.3	(1.7±0.2) × 10^{-1}
P	3.4±0.5	2.7±0.5	(1.0±0.2) × 10^{-1}	(2.6±0.3) × 10	(3.3±0.7) × 10^{-5}
F	7.9±0.7	5.7±0.8	1.8±0.2	(8.5±1.2) × 10	(2.7±0.4) × 10^{-2}
Y	(1.0±0.1) × 10	9.3±1.7	2.3±0.6	(5.7±0.7) × 10	(4.2±1.0) × 10^{-2}
W	(2.9±0.3) × 10	(3.5±0.4) × 10	1.4±0.1	(3.2±0.5) × 10	
S	(1.9±0.3) × 10^{2}	(1.3±0.2) × 10^{2}	(2.8±0.4) × 10	(1.0±0.2) × 10	(9.7±1.5) × 10^{-2}
T	(1.2±0.3) × 10^{2}	(8.9±1.3) × 10	(1.2±0.1) × 10	(1.4±0.3) × 10	(2.5±0.5) × 10^{-2}
N	(1.3±0.2) × 10^{1}	(1.2±0.3) × 10	4.5±0.8	2.0±0.4	
Q	4.6±0.8	4.0±1.0	1.4±0.1	1.6±0.2	(3.6±0.6) × 10^{-2}
D	5.8±1.0	9.6±2.2	(5.1±0.3) × 10^{-1}	(2.0±0.2) × 10^{-1}	(2.4±0.3) × 10^{-2}
E	n.d.	n.d.	(1.3±0.2) × 10^{-1}	n.d.	(5±2) × 10^{-3}
H (WT)	1.00±0.09	1.0±0.1	1.00±0.05	1.0±0.1	1.0±0.1
K	(9.7±2.6) × 10	(1.4±0.5) × 10^{2}	(1.9±0.2) × 10^{-1}	3.4±0.4	
R	(4.2±0.4) × 10	(2.7±0.3) × 10	1.1±0.2	6.3±0.8	(2±1) × 10^{-3}

Table S4. Activity ratios k_2^{mutant}/k_2^{WT} from activity assays of spin-column purified *Rl*PMH T107X variants, after correction for protein concentration (see experimental section) and normalization to the respective wild-type enzymes. All reactions were carried out in triplicate at 30°C in 100 mM Tris-HCl, pH 7.5 + 500 mM NaCl + 100 μM $MnCl_2$. Substrate concentrations as indicated in Table S2. Errors correspond to the standard deviation from triplicate measurements.

**Amino acid X**	**Substrate**				
	**1**	**2b**	**3a**	**3c**	**4**
G	2.9±0.1	(2.7±0.2) × 10^{-1}	(1.6±0.5) × 10^{-1}	3.6±0.1	(4.8±0.3) × 10^{-1}
A	3.5±0.2	(1.5±0.1) × 10^{-1}	(4.9±1.4) × 10^{-2}	3.2±0.3	(3.3±0.2) × 10^{-1}
V	(9.3±3.3) × 10^{-2}	(2.5±0.2) × 10^{-2}	(7.9±2.5) × 10^{-2}	1.3±0.1	(2.7±0.8) × 10^{-2}
L	(3.3±0.5) × 10^{-1}	(1.6±0.2) × 10^{-2}	(1.0±0.3) × 10^{-1}	(9.4±0.3) × 10^{-1}	(1.4±0.1) × 10^{-1}
I	(6.6±0.8) × 10^{-2}	(4.1±0.3) × 10^{-2}	(1.2±0.4) × 10^{-1}	(5.6±0.3) × 10^{-1}	(2.8±1.1) × 10^{-2}
C	(4.1±0.6) × 10^{-1}	(6.4±1.2) × 10^{-2}	(8.3±2.5) × 10^{-2}	(6.5±0.3) × 10^{-1}	(1.2±0.1) × 10^{-1}
M	(6.4±0.7) × 10^{-1}	(5.2±0.4) × 10^{-2}	(1.0±0.3) × 10^{-1}	(3.5±0.1) × 10^{-1}	(7.3±0.3) × 10^{-1}
P	(1.3±0.3) × 10^{-1}		(1.0±0.3) × 10^{-2}	(1.6±0.1) × 10^{-1}	(1.0±0.8) × 10^{-2}
F	(1.5±0.8) × 10^{-1}	(1.7±0.1) × 10^{-1}	(1.4±0.4) × 10^{-1}	(8.9±0.4) × 10^{-2}	(2.3±0.1) × 10^{-1}
Y	(7.9±0.6) × 10^{-2}	(1.2±0.1) × 10^{-1}	(1.3±0.4) × 10^{-1}	(5.2±0.3) × 10^{-2}	(9.0±0.7) × 10^{-2}
W	(8.6±1.6) × 10^{-2}	(8±1) × 10^{-3}	(2.6±0.8) × 10^{-2}	(4±1) × 10^{-3}	(1.4±0.3) × 10^{-2}
S	2.7±0.2	(9.7±1.0) × 10^{-1}	(6.6±2.6) × 10^{-1}	1.6±0.2	(6.8±0.5) × 10^{-1}
T (WT)	1.00±0.01	1.00±0.06	1.0±0.3	1.00±0.04	1.00±0.04
N	2.9±0.4	(9±4) × 10^{-3}	(4.6±1.5) × 10^{-2}	(8.7±0.5) × 10^{-2}	(7.4±1.9) × 10^{-1}
Q	2.13±0.08		(2.4±0.7) × 10^{-2}	(1.4±0.1) × 10^{-1}	(6.0±0.8) × 10^{-2}
D	1.3±0.1	(1.1±0.1) × 10^{-1}	(6.3±2.2) × 10^{-2}	1.5±0.1	(2.0±0.1) × 10^{-1}
E	(1.7±0.2) × 10^{-1}	(7±1) × 10^{-3}	(1.3±0.4) × 10^{-2}	(2.6±0.1) × 10^{-1}	(1.9±0.8) × 10^{-2}
H	(6.3±0.5) × 10^{-2}		(1.2±0.4) × 10^{-1}		(6.8±0.4) × 10^{-1}
K	3.2±0.1	(4±1) × 10^{-3}	(2.6±0.8) × 10^{-2}	(3.6±0.3) × 10^{-2}	(4.0±0.5) × 10^{-2}
R	9.4±0.4	(3.3±0.4) × 10^{-2}	(4.0±1.2) × 10^{-2}	(1.5±0.2) × 10^{-2}	(2.0±0.1) × 10^{-1}

Table S5. Values for hydrophobicity (LogP) and size (volume in Å^3) for the various amino acid side chains.

Amino acid	LogP[a]	Volume[b] (Å^3)
G	-0.42	60.1
A	1.09	88.6
V	1.75	140.0
L	2.08	166.7
I	2.17	166.7
C	0.56	108.5
M	1.15	162.9
P	1.75	112.7
F	2.52	189.9
Y	2.13	193.6
W	2.06	227.8
S	-0.27	89.0
T	0.07	116.1
N	-0.96	114.1
Q	-0.30	143.8
D	-0.31	111.1
E	0.35	138.4
H	0.04	153.2
K	0.59	168.6
R	0.31	173.4

[a]Calculated for the neutral amino acid side chains using ChemBioDraw 14.0
[b]According to Zamyatin et al.[4]

Table S6. Steady-state kinetic parameters for the various reactions catalyzed by *Ak*AS (0.6 mM $MnCl_2$, substrates **1-2c**, **4**: 44 mM succinic acid, 33 mM imidazole, 33 mM diethanolamine pH 7.6, substrate **3a-3c**: 20 mM bis-tris-propane pH 7.6 + 100 mM NaCl).

		WT[a]	C53S	Ratio WT/C53S
**1**	k_{cat} (s^{-1})	(8.8±0.1) × 10^{-1}	(4.8±0.1) × 10^{-3}	(2.01±0.05) × 10^{2}
	K_M (M)	(2.5±0.1) × 10^{-3}	(1.7±0.1) × 10^{-2}	(1.5±0.1) × 10^{-1}
	k_{cat}/K_M (s^{-1} M^{-1})	(3.5±0.2) × 10^{2}	(2.6±0.2) × 10^{-1}	(1.3±0.1) × 10^{3}
**2a**	k_{cat} (s^{-1})	7.5±0.1	(4.5±0.1) × 10^{-3}	(1.65±0.05) × 10^{3}
	K_M (M)	(7.9±0.2) × 10^{-2}	(5.3±0.5) × 10^{-4}	(1.5±0.1) × 10^{2}
	k_{cat}/K_M (s^{-1} M^{-1})	(9.4±0.2) × 10^{1}	8.5±0.9	(1.1±0.1) × 10^{1}
**2b**	k_{cat} (s^{-1})	(7.4±0.3) × 10^{-1}	(3.7±0.6) × 10^{-3}	(2.0±0.3) × 10^{2}
	K_M (M)	(2.8±0.2) × 10^{-2}	(8.1±2.2) × 10^{-2}	(3.4±1.0) × 10^{-1}
	k_{cat}/K_M (s^{-1} M^{-1})	(2.6±0.3) × 10^{1}	(4.6±1.5) × 10^{-2}	(5.8±2.0) × 10^{2}
**2c**	k_{cat} (s^{-1})	2.4±0.1	(3.21±0.09) × 10^{-3}	(7.4±0.4) × 10^{2}
	K_M (M)	(3.1±0.2) × 10^{-2}	(5.7±0.7) × 10^{-4}	(5.4±0.8) × 10^{1}
	k_{cat}/K_M (s^{-1} M^{-1})	(7.7±0.7) × 10^{1}	5.6±0.7	(1.4±0.2) × 10^{1}
**3a**	k_{cat} (s^{-1})	(2.72±0.06) × 10^{1}	(3.33±0.04) × 10^{-2}	(8.2±0.2) × 10^{2}
	K_M (M)	(1.9±0.1) × 10^{-2}	(3.6±0.4) × 10^{-4}	(5.3±0.4) × 10^{1}
	k_{cat}/K_M (s^{-1} M^{-1})	(1.45±0.09) × 10^{3}	(9.4±0.5) × 10^{1}	(1.5±0.1) × 10^{1}
**3b**	k_{cat} (s^{-1})	3.2±0.1	(1.06±0.01) × 10^{-2}	(3.0±0.1) × 10^{2}
	K_M (M)	(9.1±0.6) × 10^{-2}	(4.8±0.2) × 10^{-4}	(1.9±0.2) × 10^{2}
	k_{cat}/K_M (s^{-1} M^{-1})	(3.5±0.3) × 10^{1}	(2.2±0.1) × 10^{1}	1.6±0.1
**3c**	k_{cat} (s^{-1})	>7	>9 × 10^{-2}	
	K_M (M)	>7 × 10^{-2}	>5 × 10^{-3}	
	k_{cat}/K_M (s^{-1} M^{-1})	(1.16±0.02) × 10^{2}	(5.8±0.1) × 10^{1}	2.00±0.05
**4**	k_{cat} (s^{-1})	(9.7±0.2) × 10^{1}	(2.66±0.03) × 10^{-1}	(3.66±0.08) × 10^{2}
	K_M (M)	(9.9±0.6) × 10^{-5}	(2.45±0.08) × 10^{-4}	(4.0±0.3) × 10^{-1}
	k_{cat}/K_M (s^{-1} M^{-1})	(9.9±0.6) × 10^{5}	(1.09±0.04) × 10^{3}	(9.1±0.6) × 10^{2}

[a]Data from van Loo et al.[3]

Table S7. Steady-state kinetic parameters for the various reactions catalyzed by *Rp*AS (0.6 mM $MnCl_2$, substrates **1-2c**, **4**: 44 mM succinic acid, 33 mM imidazole, 33 mM diethanolamine pH 7.2, substrate **3a-3c**: 20 mM bis-tris-propane pH 7.2 + 100 mM NaCl).

		WT[a]	C53S	Ratio WT/C53S
**1**	k_{cat} (s^{-1})	(8.19±0.09) × 10^{-2}	(1.14±0.01) × 10^{-2}	7.2±0.1
	K_M (M)	(1.11±0.04) × 10^{-4}	(1.20±0.04) × 10^{-4}	(9.2±0.5) × 10^{-1}
	k_{cat}/K_M (s^{-1} M^{-1})	(7.4±0.3) × 10^{2}	(9.5±0.4) × 10^{1}	7.8±0.4
**2a**	k_{cat} (s^{-1})	2.89±0.06	(4.13±0.08) × 10^{-1}	7.0±0.2
	K_M (M)	(7.4±0.4) × 10^{-4}	(6.3±0.4) × 10^{-4}	1.2±0.1
	k_{cat}/K_M (s^{-1} M^{-1})	(3.9±0.2) × 10^{3}	(6.5±0.4) × 10^{2}	6.0±0.5
**2b**	k_{cat} (s^{-1})	(9.6±0.2) × 10^{-2}	(1.65±0.03) × 10^{-2}	5.8±0.1
	K_M (M)	(4.7±0.3) × 10^{-4}	(7.6±0.3) × 10^{-4}	(6.1±0.5) × 10^{-1}
	k_{cat}/K_M (s^{-1} M^{-1})	(2.1±0.1) × 10^{2}	(2.2±0.1) × 10^{1}	9.5±0.8
**2c**	k_{cat} (s^{-1})	(3.10±0.07) × 10^{-1}	(8.3±0.2) × 10^{-2}	3.7±0.1
	K_M (M)	(1.82±0.09) × 10^{-3}	(6.3±0.3) × 10^{-3}	(2.9±0.2) × 10^{-1}
	k_{cat}/K_M (s^{-1} M^{-1})	(1.7±0.1) × 10^{2}	(1.33±0.07) × 10^{1}	(1.3±0.1) × 10^{1}
**3a**	k_{cat} (s^{-1})	2.85±0.03	(3.74±0.04) × 10^{-1}	7.6±0.1
	K_M (M)	(5.6±0.1) × 10^{-5}	(6.0±0.2) × 10^{-5}	(9.4±0.4) × 10^{-1}
	k_{cat}/K_M (s^{-1} M^{-1})	(5.1±0.1) × 10^{4}	(6.2±0.2) × 10^{3}	8.1±0.4
**3b**	k_{cat} (s^{-1})	2.07±0.05	(3.83±0.03) × 10^{-1}	5.4±0.1
	K_M (M)	(1.7±0.1) × 10^{-3}	(2.93±0.06) × 10^{-3}	(5.9±0.4) × 10^{-1}
	k_{cat}/K_M (s^{-1} M^{-1})	(1.20±0.09) × 10^{3}	(1.31±0.03) × 10^{2}	9.2±0.7
**3c**	k_{cat} (s^{-1})	(3.55±0.08) × 10^{-1}	(7.8±0.4) × 10^{-2}	4.5±0.3
	K_M (M)	(5.3±0.4) × 10^{-3}	(8.4±0.8) × 10^{-3}	(6.4±0.8) × 10^{-1}
	k_{cat}/K_M (s^{-1} M^{-1})	(6.6±0.5) × 10^{1}	9.3±1.0	7.1±1.0
**4**	k_{cat} (s^{-1})	(1.27±0.02) × 10^{1}	1.84±0.02	6.9±0.1
	K_M (M)	(2.2±0.1) × 10^{-6}	(3.2±0.1) × 10^{-6}	(6.8±0.5) × 10^{-1}
	k_{cat}/K_M (s^{-1} M^{-1})	(5.8±0.3) × 10^{6}	(5.8±0.2) × 10^{5}	(1.01±0.07) × 10^{1}

[a]Data from van Loo et al.[3]

Table S8. Steady-state kinetic parameters for the various reactions catalyzed by *Sa*AS (0.6 mM $MnCl_2$, substrates **1-2c**, **4**: 44 mM succinic acid, 33 mM imidazole, 33 mM diethanolamine pH 7.6, substrate **3a-3c**: 20 mM bis-tris-propane pH 7.6 + 100 mM NaCl).

		WT[a]	C49S	Ratio WT/C49S
**1**	k_{cat} (s^{-1})	(2.06±0.03) × 10^{-1}	(1.35±0.01) × 10^{-1}	1.53±0.03
	K_M (M)	(1.52±0.05) × 10^{-2}	(9.9±0.3) × 10^{-3}	1.53±0.07
	k_{cat}/K_M (s^{-1} M^{-1})	(1.36±0.05) × 10^{1}	(1.36±0.05) × 10^{1}	1.00±0.05
**2a**	k_{cat} (s^{-1})	4.48±0.03	3.19±0.02	1.41±0.01
	K_M (M)	(4.5±0.1) × 10^{-3}	(3.35±0.08) × 10^{-3}	1.34±0.05
	k_{cat}/K_M (s^{-1} M^{-1})	(1.00±0.03) × 10^{3}	(9.5±0.2) × 10^{2}	1.05±0.04
**2b**	k_{cat} (s^{-1})	(4.6±0.3) × 10^{-1}	(3.28±0.05) × 10^{-1}	1.40±0.09
	K_M (M)	(6.0±0.5) × 10^{-2}	(3.8±0.1) × 10^{-2}	1.6±0.1
	k_{cat}/K_M (s^{-1} M^{-1})	7.7±0.8	8.8±0.3	(8.8±0.9) × 10^{-1}
**2c**	k_{cat} (s^{-1})	(9.7±0.1) × 10^{-2}	(7.3±0.2) × 10^{-2}	1.33±0.04
	K_M (M)	(6.6±0.2) × 10^{-3}	(6.4±0.5) × 10^{-3}	1.04±0.08
	k_{cat}/K_M (s^{-1} M^{-1})	(1.47±0.06) × 10^{1}	(1.15±0.09) × 10^{1}	1.3±0.1
**3a**	k_{cat} (s^{-1})	1.51±0.02	(6.98±0.08) × 10^{-1}	2.17±0.04
	K_M (M)	(1.31±0.05) × 10^{-4}	(3.3±0.1) × 10^{-5}	4.0±0.2
	k_{cat}/K_M (s^{-1} M^{-1})	(1.16±0.04) × 10^{4}	(2.14±0.09) × 10^{4}	(5.4±0.3) × 10^{-1}
**3b**	k_{cat} (s^{-1})	(5.57±0.06) × 10^{-1}	(3.45±0.04) × 10^{-1}	1.61±0.03
	K_M (M)	(5.3±0.2) × 10^{-4}	(6.7±0.2) × 10^{-4}	(8.0±0.4) × 10^{-1}
	k_{cat}/K_M (s^{-1} M^{-1})	(1.04±0.04) × 10^{3}	(5.2±0.2) × 10^{2}	2.0±0.1
**3c**	k_{cat} (s^{-1})	(3.79±0.04) × 10^{-1}	(1.27±0.02) × 10^{-1}	2.98±0.06
	K_M (M)	(1.38±0.06) × 10^{-3}	(1.35±0.05) × 10^{-3}	1.02±0.06
	k_{cat}/K_M (s^{-1} M^{-1})	(2.8±0.1) × 10^{2}	(9.4±0.4) × 10^{1}	2.9±0.2
**4**	k_{cat} (s^{-1})	(2.00±0.04) × 10^{2}	(9.3±0.2) × 10^{1}	2.15±0.06
	K_M (M)	(2.8±0.1) × 10^{-4}	(2.4±0.1) × 10^{-4}	1.17±0.08
	k_{cat}/K_M (s^{-1} M^{-1})	(7.0±0.4) × 10^{5}	(3.8±0.2) × 10^{5}	1.8±0.1

[a]Data from van Loo et al.[3]

Table S9. Steady-state kinetic parameters for the various reactions catalyzed by *Sp*AS1 (0.2 mM $MnCl_2$, substrates **1-2c**, **4**: 44 mM succinic acid, 33 mM imidazole, 33 mM diethanolamine pH 7.5, substrate **3a-3c**: 20 mM bis-tris-propane pH 7.5 + 100 mM NaCl).

		WT[a]	C53S	Ratio WT/C53S
**1**	k_{cat} (s^{-1})	(2.09±0.05) × 10^{-2}	(1.29±0.04) × 10^{-4}	(1.62±0.06) × 10^{1}
	K_M (M)	(3.1±0.2) × 10^{-2}	(8.1±0.7) × 10^{-3}	3.8±0.5
	k_{cat}/K_M (s^{-1} M^{-1})	(6.8±0.5) × 10^{-1}	(1.6±0.2) × 10^{-1}	4.3±0.6
**2a**	k_{cat} (s^{-1})	>1.5 × 10^{-2}	(1.08±0.03) × 10^{-3}	[b]
	K_M (M)	>7 × 10^{-2}	(1.42±0.08) × 10^{-1}	[b]
	k_{cat}/K_M (s^{-1} M^{-1})	(2.09±0.03) × 10^{-1}	(7.8±0.5) × 10^{-3}	(2.8±0.2) × 10^{1}
**2b**	k_{cat} (s^{-1})	(6.3±0.3) × 10^{-3}	(3.8±0.2) × 10^{-4}	(1.6±0.1) × 10^{1}
	K_M (M)	(1.21±0.09) × 10^{-1}	(7.0±1.1) × 10^{-3}	(1.7±0.3) × 10^{1}
	k_{cat}/K_M (s^{-1} M^{-1})	(5.2±0.5) × 10^{-2}	(5.5±0.9) × 10^{-2}	1.0±0.2
**2c**	k_{cat} (s^{-1})	>1.5 × 10^{-2}	>2 × 10^{-4}	[b]
	K_M (M)	>5 × 10^{-2}	>2 × 10^{-2}	[b]
	k_{cat}/K_M (s^{-1} M^{-1})	(9.1±3.1) × 10^{-1}	(7.53±0.09) × 10^{-3}	(1.2±0.4) × 10^{2}
**3a**	k_{cat} (s^{-1})	(5.1±0.2) × 10^{-1}	(7.7±0.2) × 10^{-3}	(6.6±0.3) × 10^{1}
	K_M (M)	(8.4±0.5) × 10^{-2}	(1.6±0.2) × 10^{-2}	(5.4±0.7) × 10^{1}
	k_{cat}/K_M (s^{-1} M^{-1})	6.1±0.4	(5.0±0.6) × 10^{-1}	(1.2±0.2) × 10^{1}
**3b**	k_{cat} (s^{-1})	>5 × 10^{-4}	(2.3±0.1) × 10^{-4}	[b]
	K_M (M)	>3 × 10^{-2}	(7.2±1.0) × 10^{-2}	[b]
	k_{cat}/K_M (s^{-1} M^{-1})	(1.72±0.06) × 10^{-2}	(3.2±0.5) × 10^{-3}	5.4±0.8
**3c**	k_{cat} (s^{-1})	>1 × 10^{-1}	(7.3±0.9) × 10^{-3}	[b]
	K_M (M)	>6.5 × 10^{-2}	(1.9±0.3) × 10^{-2}	[b]
	k_{cat}/K_M (s^{-1} M^{-1})	6.7±0.9	(3.8±0.8) × 10^{-1}	(1.8±0.5) × 10^{1}
**4**	k_{cat} (s^{-1})	(2.04±0.07) × 10^{1}	(3.0±0.7) × 10^{-1}	(6.7±1.7) × 10^{1}
	K_M (M)	(4.0±0.3) × 10^{-3}	(5.0±1.9) × 10^{-3}	(8.2±3.2) × 10^{-1}
	k_{cat}/K_M (s^{-1} M^{-1})	(5.0±0.4) × 10^{3}	(6.1±2.8) × 10^{1}	(8.2±3.8) × 10^{1}

[a]Data from van Loo et al.[3]
[b]Saturating conditions could not be reached for this substrate with either the wild-type or the mutant enzyme or both, therefore k_{cat}/K_M was determined using equation 3 and only lower limits for k_{cat} and K_M could be obtained.

Table S10. Steady-state kinetic parameters for the various reactions catalyzed by *Sp*AS2 (0.6 mM $MnCl_2$, substrates **1-2c**, **4**: 44 mM succinic acid, 33 mM imidazole, 33 mM diethanolamine pH 8.0, substrate **3a-3c**: 20 mM bis-tris-propane pH 8.0 + 100 mM NaCl).

		WT[a]	C49S	Ratio WT/C49S
**1**	k_{cat} (s^{-1})	(9.3±0.2) × 10^{-2}	(1.16±0.01) × 10^{-2}	8.0±0.2
	K_M (M)	(1.65±0.07) × 10^{-2}	(1.41±0.06) × 10^{-2}	1.17±0.07
	k_{cat}/K_M (s^{-1} M^{-1})	5.6±0.3	(8.2±0.4) × 10^{-1}	6.9±0.5
**2a**	k_{cat} (s^{-1})	6.0±0.3	(4.7±0.2) × 10^{-1}	(1.28±0.07) × 10^{1}
	K_M (M)	(5.1±0.5) × 10^{-2}	(1.1±0.4) × 10^{-2}	4.6±1.8
	k_{cat}/K_M (s^{-1} M^{-1})	(1.2±0.1) × 10^{2}	(4.3±1.7) × 10^{1}	2.8±1.1
**2b**	k_{cat} (s^{-1})	(1.97±0.08) × 10^{-1}	(2.50±0.09) × 10^{-2}	7.9±0.4
	K_M (M)	(4.5±0.3) × 10^{-2}	(5.0±0.3) × 10^{-2}	(9.1±0.9) × 10^{-1}
	k_{cat}/K_M (s^{-1} M^{-1})	4.4±0.4	(5.0±0.4) × 10^{-1}	8.7±1.0
**2c**	k_{cat} (s^{-1})	(1.6±0.1) × 10^{-1}	(4.5±0.2) × 10^{-2}	3.6±0.3
	K_M (M)	(3.8±0.5) × 10^{-2}	(1.22±0.07) × 10^{-1}	(3.1±0.4) × 10^{-1}
	k_{cat}/K_M (s^{-1} M^{-1})	4.3±0.6	(3.7±0.2) × 10^{-1}	(1.2±0.2) × 10^{1}
**3a**	k_{cat} (s^{-1})	2.13±0.02	(1.35±0.01) × 10^{-1}	(1.57±0.02) × 10^{1}
	K_M (M)	(1.94±0.07) × 10^{-3}	(1.41±0.05) × 10^{-4}	(1.38±0.07) × 10^{1}
	k_{cat}/K_M (s^{-1} M^{-1})	(1.09±0.04) × 10^{3}	(9.6±0.4) × 10^{2}	1.14±0.06
**3b**	k_{cat} (s^{-1})	1.17±0.01	(1.11±0.01) × 10^{-1}	(1.05±0.01) × 10^{1}
	K_M (M)	(3.7±0.1) × 10^{-3}	(6.4±0.1) × 10^{-3}	(5.9±0.2) × 10^{-1}
	k_{cat}/K_M (s^{-1} M^{-1})	(3.14±0.09) × 10^{2}	(1.74±0.04) × 10^{1}	(1.80±0.07) × 10^{1}
**3c**	k_{cat} (s^{-1})	1.09±0.01	(9.79±0.09) × 10^{-2}	(1.12±0.02) × 10^{1}
	K_M (M)	(2.7±0.1) × 10^{-3}	(4.0±0.1) × 10^{-1}	(6.9±0.3) × 10^{-1}
	k_{cat}/K_M (s^{-1} M^{-1})	(4.0±0.2) × 10^{2}	(2.48±0.07) × 10^{1}	(1.62±0.08) × 10^{1}
**4**	k_{cat} (s^{-1})	(4.2±0.4) × 10^{2}	(3.8±0.2) × 10^{1}	(1.1±0.1) × 10^{1}
	K_M (M)	(2.0±0.3) × 10^{-3}	(1.7±0.2) × 10^{-3}	1.1±0.2
	k_{cat}/K_M (s^{-1} M^{-1})	(2.1±0.4) × 10^{5}	(2.2±0.3) × 10^{4}	9.6±2.0

[a]Data from van Loo et al.[3]

Table S11. Steady-state kinetic parameters for the various reactions catalyzed by *Ak*PMH (0.6 mM $MnCl_2$, substrates **1-2c**, **4**: 44 mM succinic acid, 33 mM imidazole, 33 mM diethanolamine pH 6.8, substrate **3a-3c**: 20 mM bis-tris-propane pH 6.8 + 100 mM NaCl).

		WT	C52S	Ratio WT/C52S
**1**	k_{cat} (s^{-1})	(8.8±0.1) × 10^{-2}	(2.50±0.02) × 10^{-3}	(3.52±0.07) × 10^{1}
	K_M (M)	(2.40±0.08) × 10^{-3}	(8.4±0.4) × 10^{-5}	(2.9±0.2) × 10^{1}
	k_{cat}/K_M (s^{-1} M^{-1})	(3.6±0.1) × 10^{1}	(3.0±0.1) × 10^{1}	1.23±0.07
**2a**	k_{cat} (s^{-1})	4.8±0.1	(2.0±0.8) × 10^{-2}	(2.4±1.0) × 10^{2}
	K_M (M)	(1.47±0.09) × 10^{-2}	(3.4±1.7) × 10^{-2}	(4.4±2.2) × 10^{-1}
	k_{cat}/K_M (s^{-1} M^{-1})	(3.3±0.2) × 10^{2}	(6.1±3.9) × 10^{-1}	(5.4±3.5) × 10^{2}
**2b**	k_{cat} (s^{-1})	2.15±0.04	(5.44±0.07) × 10^{-3}	(3.96±0.08) × 10^{2}
	K_M (M)	(5.0±0.2) × 10^{-3}	(7.5±0.3) × 10^{-4}	6.7±0.4
	k_{cat}/K_M (s^{-1} M^{-1})	(4.3±0.2) × 10^{2}	7.3±0.3	(5.9±0.4) × 10^{1}
**2c**	k_{cat} (s^{-1})	1.74±0.08	(2.62±0.07) × 10^{-3}	(6.7±0.3) × 10^{2}
	K_M (M)	(1.8±0.1) × 10^{-3}	(7.1±0.9) × 10^{-4}	2.5±0.4
	k_{cat}/K_M (s^{-1} M^{-1})	(9.8±0.8) × 10^{2}	3.7±0.5	(2.7±0.4) × 10^{2}
**3a**	k_{cat} (s^{-1})	(1.40±0.04) × 10^{1}	(6.23±0.06) × 10^{-3}	(2.26±0.07) × 10^{3}
	K_M (M)	(2.9±0.2) × 10^{-3}	(1.50±0.07) × 10^{-3}	2.0±0.2
	k_{cat}/K_M (s^{-1} M^{-1})	(4.8±0.3) × 10^{3}	4.2±0.2	(1.15±0.09) × 10^{3}
**3b**	k_{cat} (s^{-1})	9.0±0.2	(3.62±0.06) × 10^{-3}	(2.49±0.06) × 10^{3}
	K_M (M)	(1.45±0.08) × 10^{-3}	(1.8±0.1) × 10^{-4}	8.3±0.7
	k_{cat}/K_M (s^{-1} M^{-1})	(6.2±0.4) × 10^{3}	(2.1±0.1) × 10^{1}	(3.0±0.3) × 10^{2}
**3c**	k_{cat} (s^{-1})	(3.40±0.08) × 10^{-1}	(3.18±0.05) × 10^{-3}	(1.07±0.03) × 10^{2}
	K_M (M)	(1.06±0.08) × 10^{-4}	(7.9±0.5) × 10^{-5}	1.3±0.1
	k_{cat}/K_M (s^{-1} M^{-1})	(3.2±0.2) × 10^{3}	(4.0±0.3) × 10^{1}	(7.9±0.8) × 10^{1}
**4**	k_{cat} (s^{-1})	(1.75±0.06) × 10^{-1}	(1.22±0.05) × 10^{-4}	(1.44±0.08) × 10^{3}
	K_M (M)	(1.0±0.2) × 10^{-2}	(3.6±0.2) × 10^{-2}	(2.9±0.5) × 10^{-1}
	k_{cat}/K_M (s^{-1} M^{-1})	(1.7±0.3) × 10^{1}	(3.4±0.3) × 10^{-3}	(5.0±0.8) × 10^{3}

[a]Data from van Loo et al.[3]

Table S12. Steady-state kinetic parameters for the various reactions catalyzed by *Bc*PMH (0.6 mM $MnCl_2$, substrates **1-2c**, **4**: 44 mM succinic acid, 33 mM imidazole, 33 mM diethanolamine pH 7.5, substrate **3a-3c**: 20 mM bis-tris-propane pH 7.5 + 100 mM NaCl).

		WT[a]	C57S	Ratio WT/C57S
**1**	k_{cat} (s^{-1})	(2.49±0.02) × 10^{-2}	(1.14±0.01) × 10^{-3}	(2.19±0.03) × 10^{1}
	K_M (M)	(3.03±0.09) × 10^{-4}	(2.4±0.1) × 10^{-4}	1.27±0.08
	k_{cat}/K_M (s^{-1} M^{-1})	(8.2±0.3) × 10^{1}	4.8±0.3	(1.7±0.1) × 10^{1}
**2a**	k_{cat} (s^{-1})	(1.97±0.02) × 10^{1}	(9.84±0.09) × 10^{-1}	(2.00±0.02) × 10^{1}
	K_M (M)	(1.16±0.03) × 10^{-3}	(7.5±0.3) × 10^{-4}	1.55±0.06
	k_{cat}/K_M (s^{-1} M^{-1})	(1.70±0.04) × 10^{4}	(1.32±0.05) × 10^{3}	(1.28±0.06) × 10^{1}
**2b**	k_{cat} (s^{-1})	8.8±0.1	(4.03±0.09) × 10^{-1}	(2.18±0.06) × 10^{1}
	K_M (M)	(3.5±0.2) × 10^{-4}	(2.5±0.2) × 10^{-4}	1.4±0.1
	k_{cat}/K_M (s^{-1} M^{-1})	(2.5±0.1) × 10^{4}	(1.6±0.2) × 10^{3}	(1.6±0.2) × 10^{1}
**2c**	k_{cat} (s^{-1})	1.79±0.01	(1.35±0.02) × 10^{-1}	(1.32±0.02) × 10^{1}
	K_M (M)	(3.05±0.07) × 10^{-5}	(1.7±0.1) × 10^{-5}	1.8±0.1
	k_{cat}/K_M (s^{-1} M^{-1})	(5.9±0.1) × 10^{4}	(8.2±0.5) × 10^{3}	7.2±0.5
**3a**	k_{cat} (s^{-1})	(1.07±0.01) × 10^{2}	2.17±0.05	(4.9±0.1) × 10^{1}
	K_M (M)	(8.0±0.3) × 10^{-3}	(1.8±0.1) × 10^{-3}	4.6±0.4
	k_{cat}/K_M (s^{-1} M^{-1})	(1.34±0.05) × 10^{4}	(1.24±0.09) × 10^{3}	(1.08±0.09) × 10^{1}
**3b**	k_{cat} (s^{-1})	(1.78±0.01) × 10^{1}	1.08±0.01	(1.64±0.02) × 10^{1}
	K_M (M)	(1.89±0.03) × 10^{-4}	(1.56±0.05) × 10^{-4}	1.21±0.04
	k_{cat}/K_M (s^{-1} M^{-1})	(9.4±0.2) × 10^{4}	(6.9±0.2) × 10^{3}	(1.36±0.05) × 10^{1}
**3c**	k_{cat} (s^{-1})	2.16±0.03	(1.30±0.01) × 10^{-1}	(1.66±0.02) × 10^{1}
	K_M (M)	(5.1±0.3) × 10^{-5}	(3.7±0.1) × 10^{-5}	1.4±0.1)
	k_{cat}/K_M (s^{-1} M^{-1})	(4.2±0.3) × 10^{4}	(3.5±0.1) × 10^{3}	(1.19±0.08) × 10^{1}
**4**	k_{cat} (s^{-1})	(1.09±0.03) × 10^{-1}	(3.17±0.07) × 10^{-3}	(3.4±0.1) × 10^{1}
	K_M (M)	(7.9±0.4) × 10^{-2}	(5.5±0.2) × 10^{-2}	1.4±0.1
	k_{cat}/K_M (s^{-1} M^{-1})	1.38±0.08	(5.8±0.3) × 10^{-2}	(2.4±0.2) × 10^{1}

[a]Data from van Loo et al.[3]

Table S13. Steady-state kinetic parameters for the various reactions catalyzed by *Rl*PMH (0.6 mM $MnCl_2$, substrates **1-2c**, **4**: 44 mM succinic acid, 33 mM imidazole, 33 mM diethanolamine pH 7.5, substrate **3a-3c**: 20 mM bis-tris-propane pH 7.5 + 100 mM NaCl).

		WT[a]	C57S	Ratio WT/C57S
**1**	k_{cat} (s^{-1})	(9.2±0.1) × 10^{-2}	(5.6±0.1) × 10^{-3}	(1.65±0.04) × 10^{1}
	K_M (M)	(5.2±0.3) × 10^{-4}	(3.2±0.2) × 10^{-4}	1.6±0.2
	k_{cat}/K_M (s^{-1} M^{-1})	(1.7±0.1) × 10^{2}	(1.8±0.1) × 10^{1}	(1.0±0.1) × 10^{1}
**2a**	k_{cat} (s^{-1})	(1.10±0.05) × 10^{1}	(5.18±0.06) × 10^{-1}	(2.1±0.1) × 10^{1}
	K_M (M)	(1.3±0.2) × 10^{-3}	(1.15±0.04) × 10^{-3}	1.2±0.2
	k_{cat}/K_M (s^{-1} M^{-1})	(8.4±1.3) × 10^{3}	(4.5±0.2) × 10^{2}	(1.9±0.3) × 10^{1}
**2b**	k_{cat} (s^{-1})	(1.29±0.01) × 10^{1}	(3.68±0.05) × 10^{-3}	(3.50±0.06) × 10^{1}
	K_M (M)	(1.5±0.1) × 10^{-3}	(5.8±0.4) × 10^{-2}	2.6±0.3
	k_{cat}/K_M (s^{-1} M^{-1})	(8.9±0.6) × 10^{3}	(6.4±0.5) × 10^{2}	(1.4±0.1) × 10^{1}
**2c**	k_{cat} (s^{-1})	1.5±0.1	(6.5±0.1) × 10^{-2}	(2.3±0.2) × 10^{1}
	K_M (M)	(2.8±0.6) × 10^{-5}	(1.8±0.1) × 10^{-5}	1.5±0.3
	k_{cat}/K_M (s^{-1} M^{-1})	(5.2±1.2) × 10^{4}	(3.5±0.2) × 10^{3}	(1.5±0.4) × 10^{1}
**3a**	k_{cat} (s^{-1})	(1.18±0.06) × 10^{2}	3.60±0.08	(3.3±0.2) × 10^{1}
	K_M (M)	(1.3±0.1) × 10^{-2}	(1.8±0.1) × 10^{-3}	7.4±0.7
	k_{cat}/K_M (s^{-1} M^{-1})	(9.1±0.8) × 10^{3}	(2.0±0.1) × 10^{3}	4.5±0.5
**3b**	k_{cat} (s^{-1})	(3.16±0.06) × 10^{1}	1.84±0.02	(1.72±0.04) × 10^{1}
	K_M (M)	(1.8±0.1) × 10^{-3}	(1.67±0.08) × 10^{-4}	(1.08±0.08) × 10^{1}
	k_{cat}/K_M (s^{-1} M^{-1})	(1.8±0.1) × 10^{4}	(1.10±0.05) × 10^{4}	1.6±0.1
**3c**	k_{cat} (s^{-1})	(1.83±0.02) × 10^{1}	(4.58±0.06) × 10^{-1}	(4.00±0.07) × 10^{1}
	K_M (M)	(1.2±0.1) × 10^{-3}	(7.3±0.4) × 10^{-5}	(1.7±0.2) × 10^{1}
	k_{cat}/K_M (s^{-1} M^{-1})	(1.5±0.1) × 10^{4}	(6.3±0.4) × 10^{3}	2.4±0.2
**4**	k_{cat} (s^{-1})	>2 × 10^{-1}	(1.86±0.04) × 10^{-2}	[b]
	K_M (M)	>8 × 10^{-2}	(2.2±0.3) × 10^{-2}	[b]
	k_{cat}/K_M (s^{-1} M^{-1})	2.8±0.1	(8.6±1.2) × 10^{-1}	3.3±0.5

[a]Data from van Loo et al.[3]
[b]Saturating conditions could not be reached for this substrate with either the wild-type or the mutant enzyme or both, therefore k_{cat}/K_M was determined using equation 3 and only lower limits for k_{cat} and K_M could be obtained.

Table S14. Steady-state kinetic parameters for the various reactions catalyzed by *Sp*PMH (5 mM $MnCl_2$, substrates **1**-**2c**: 44 mM succinic acid, 33 mM imidazole, 33 mM diethanolamine pH 6.0, substrate **3a**-**3c**: 20 mM bis-tris-propane pH 6.0 + 100 mM NaCl, substrate **4**: 44 mM succinic acid, 33 mM imidazole, 33 mM diethanolamine pH 7.5).

		WT[a]	C53S	Ratio WT/C53S
**1**	k_{cat} (s^{-1})	$(4.7±0.2) \times 10^{-2}$	n.d.	n.d.
	K_M (M)	$(1.8±0.3) \times 10^{-3}$	n.d.	b
	k_{cat}/K_M (s^{-1} M^{-1})	$(2.6±0.5) \times 10^{1}$	n.d.	b
**2a**	k_{cat} (s^{-1})	>4	$>5 \times 10^{-5}$	b
	K_M (M)	$>4.5 \times 10^{-2}$	$>1 \times 10^{-1}$	b
	k_{cat}/K_M (s^{-1} M^{-1})	$(8.8±0.2) \times 10^{1}$	$(4.97±0.09) \times 10^{-2}$	$(1.77±0.05) \times 10^{2}$
**2b**	k_{cat} (s^{-1})	3.9±0.4	$(3.19±0.09) \times 10^{-2}$	$(1.2±0.1) \times 10^{2}$
	K_M (M)	$(2.8±0.5) \times 10^{-2}$	$(3.5±0.2) \times 10^{-2}$	$(7.9±1.5) \times 10^{-1}$
	k_{cat}/K_M (s^{-1} M^{-1})	$(1.4±0.3) \times 10^{2}$	$(9.0±0.6) \times 10^{-2}$	$(1.5±0.3) \times 10^{2}$
**2c**	k_{cat} (s^{-1})	$>7 \times 10^{-1}$	n.d.	n.d.
	K_M (M)	$>4 \times 10^{-2}$	n.d.	n.d.
	k_{cat}/K_M (s^{-1} M^{-1})	$(1.0±0.4) \times 10^{2}$	n.d.	n.d.
**3a**	k_{cat} (s^{-1})	>4.5	n.d.	n.d.
	K_M (M)	$>6 \times 10^{-2}$	n.d.	n.d.
	k_{cat}/K_M (s^{-1} M^{-1})	$(1.2±0.3) \times 10^{2}$	n.d.	n.d.
**3b**	k_{cat} (s^{-1})	$>1.2 \times 10^{1}$	n.d.	n.d.
	K_M (M)	$>8.5 \times 10^{-2}$	n.d.	n.d.
	k_{cat}/K_M (s^{-1} M^{-1})	$(1.44±0.03) \times 10^{2}$	n.d.	n.d.
**3c**	k_{cat} (s^{-1})	>1.5	n.d.	n.d.
	K_M (M)	$>2.5 \times 10^{-2}$	n.d.	n.d.
	k_{cat}/K_M (s^{-1} M^{-1})	$(5.5±0.1) \times 10^{1}$	n.d.	n.d.
**4**	k_{cat} (s^{-1})	$>2 \times 10^{-3}$	$>1 \times 10^{-5}$	b
	K_M (M)	$>7 \times 10^{-2}$	$>7 \times 10^{-2}$	b
	k_{cat}/K_M (s^{-1} M^{-1})	$(1.42±0.03) \times 10^{-2}$	$(1.48±0.04) \times 10^{-3}$	9.6±0.3

[a]Data from van Loo et al.[3]

[b]Saturating conditions could not be reached for this substrate with either the wild-type or the mutant enzyme or both, therefore k_{cat}/K_M was determined using equation 3 and only lower limits for k_{cat} and K_M could be obtained.

Table S15. Primers used for site-saturating mutagenesis of H103 in *Sp*AS1

Mutation	primers[a]	
H103G	sense	5'-CTGATCGGGAAAACC**ggc**ATGCGCCCTGATCTG-3'
	anti-sense	5'-CAGATCAGGGCGCAT**gcc**GGTTTTCCCGATCAG-3'
H103A	sense	5'-CTGATCGGGAAAACC**gcg**ATGCGCCCTGATCTG-3'
	anti-sense	5'-CAGATCAGGGCGCAT**cgc**GGTTTTCCCGATCAG-3'
H103V	sense	5'-CTGATCGGGAAAACC**gtg**ATGCGCCCTGATCTG-3'
	anti-sense	5'-CAGATCAGGGCGCAT**cac**GGTTTTCCCGATCAG-3'
H103L	sense	5'-GATCGGGAAAACC**ttg**ATGCGCCCTGATC-3'
	anti-sense	5'-GATCAGGGCGCAT**caa**GGTTTTCCCGATC-3'
H103I	sense	5'-CTGATCGGGAAAACC**atT**ATGCGCCCTGATC-3'
	anti-sense	5'-GATCAGGGCGCAT**Aat**GGTTTTCCCGATCAG-3'
H103C	sense	5'-CTGATCGGGAAAACC**tgc**ATGCGCCCTGATCTG-3'
	anti-sense	5'-CAGATCAGGGCGCAT**gca**GGTTTTCCCGATCAG-3'
H103M	sense	5'-CCTGATCGGGAAAACC**atg**ATGCGCCCTGATCTG-3'
	anti-sense	5'-CAGATCAGGGCGCAT**cat**GGTTTTCCCGATCAGG-3'
H103P	sense	5'-GATCGGGAAAACC**Ccg**ATGCGCCCTGATC-3'
	anti-sense	5'-GATCAGGGCGCAT**cgG**GGTTTTCCCGATC-3'
H103F	sense	5'-CTGATCGGGAAAACC**ttc**ATGCGCCCTGATC-3'
	anti-sense	5'-GATCAGGGCGCAT**gaa**GGTTTTCCCGATCAG-3'
H103Y	sense	5'-CTGATCGGGAAAACC**tAc**ATGCGCCCTGATC-3'
	anti-sense	5'-GATCAGGGCGCAT**gTa**GGTTTTCCCGATCAG-3'
H103W	sense	5'-CTGATCGGGAAAACC**tgg**ATGCGCCCTGATCTG-3'
	anti-sense	5'-CAGATCAGGGCGCAT**cca**GGTTTTCCCGATCAG-3'
H103S	sense	5'-CTGATCGGGAAAACC**tcg**ATGCGCCCTGATCTG-3'
	anti-sense	5'-CAGATCAGGGCGCAT**cga**GGTTTTCCCGATCAG-3'
H103T	sense	5'-CTGATCGGGAAAACC**acT**ATGCGCCCTGATCTG-3'
	anti-sense	5'-CAGATCAGGGCGCAT**Agt**GGTTTTCCCGATCAG-3'
H103N	sense	5'-CTGATCGGGAAAACC**aAT**ATGCGCCCTGATC-3'
	anti-sense	5'-GATCAGGGCGCAT**ATt**GGTTTTCCCGATCAG-3'
H103Q	sense	5'-CGGGAAAACC**CAg**ATGCGCCCTGATC-3'
	anti-sense	5'-GATCAGGGCGCAT**cTG**GGTTTTCCCG-3'
H103D	sense	5'-GATCGGGAAAACC**gAT**ATGCGCCCTG-3'
	anti-sense	5'-CAGGGCGCAT**ATc**GGTTTTCCCGATC-3'
H103E	sense	5'-CCTGATCGGGAAAACC**gAg**ATGCGCCCTGATCTG-3'
	anti-sense	5'-CAGATCAGGGCGCAT**cTc**GGTTTTCCCGATCAGG-3'
H103K	sense	5'-CCTGATCGGGAAAACC**aAg**ATGCGCCCTGATCTGG-3'
	anti-sense	5'-CCAGATCAGGGCGCAT**cTt**GGTTTTCCCGATCAGG-3'
H103R	sense	5'-GATCGGGAAAACC**Cgg**ATGCGCCCTGATC-3'
	anti-sense	5'-GATCAGGGCGCAT**ccG**GGTTTTCCCGATC-3'

[a]Mismatch bases in lower case, mutated codon in boldface.

Table S16. Primers used for site-saturation mutagenesis of T107 in *Rl*PMH

Mutation	Primer[a]	
T107G	sense	5'-GATCGGCTATACG**ggG**ACGGTGCCGGATC-3'
	anti-sense	5'-GATCCGGCACCGT**Ccc**CGTATAGCCGATC-3'
T107A	sense	5'-GATCGGCTATACG**gCG**ACGGTGCCGG-3'
	anti-sense	5'-CCGGCACCGT**CGc**CGTATAGCCGATC-3'
T107V	sense	5'-GATCGGCTATACG**gtc**ACGGTGCCGGATC-3'
	anti-sense	5'-GATCCGGCACCGT**gac**CGTATAGCCGATC-3'
T107L	sense	5'-GATCGGCTATACG**ttG**ACGGTGCCGGATC-3'
	anti-sense	5'-GATCCGGCACCGT**Caa**CGTATAGCCGATC-3'
T107I	sense	5'-CTGATCGGCTATACG**Att**ACGGTGCCGGATCCGCG-3'
	anti-sense	5'-CGCGGATCCGGCACCGT**aaT**CGTATAGCCGATCAG-3'
T107C	sense	5'-CTGATCGGCTATACG**tgc**ACGGTGCCGGATCC-3'
	anti-sense	5'-GGATCCGGCACCGT**gca**CGTATAGCCGATCAG-3'
T107M	sense	5'-GATCGGCTATACG**AtG**ACGGTGCCGGATC-3'
	anti-sense	5'-GATCCGGCACCGT**CaT**CGTATAGCCGATC-3'
T107P	sense	5'-CTGATCGGCTATACG**cCG**ACGGTGCCGGATC-3'
	anti-sense	5'-GATCCGGCACCGT**CGg**CGTATAGCCGATCAG-3'
T107F	sense	5'-CTGATCGGCTATACG**ttc**ACGGTGCCGGATCCG-3'
	anti-sense	5'-CGGATCCGGCACCGT**gaa**CGTATAGCCGATCAG-3'
T107Y	sense	5'-CTGATCGGCTATACG**tac**ACGGTGCCGGATCCG-3'
	anti-sense	5'-CGGATCCGGCACCGT**gta**CGTATAGCCGATCAG-3'
T107W	sense	5'-CTGATCGGCTATACG**tgG**ACGGTGCCGGATCC-3'
	anti-sense	5'-GGATCCGGCACCGT**Cca**CGTATAGCCGATCAG-3'
T107S	sense	5'-GATCGGCTATACG**tCc**ACGGTGCCGGATC-3'
	anti-sense	5'-GATCCGGCACCGT**gGa**CGTATAGCCGATC-3'
T107N	sense	5'-CTGATCGGCTATACG**Aat**ACGGTGCCGGATCCGCG-3'
	anti-sense	5'-CGCGGATCCGGCACCGT**atT**CGTATAGCCGATCAG-3'
T107Q	sense	5'-CTGATCGGCTATACG**caG**ACGGTGCCGGATCC-3'
	anti-sense	5'-GGATCCGGCACCGT**Ctg**CGTATAGCCGATCAG-3'
T107D	sense	5'-CTGATCGGCTATACG**gac**ACGGTGCCGGATCC-3'
	anti-sense	5'-GGATCCGGCACCGT**gtc**CGTATAGCCGATCAG-3'
T107E	sense	5'-CTGATCGGCTATACG**gaG**ACGGTGCCGGATCC-3'
	anti-sense	5'-GGATCCGGCACCGT**Ctc**CGTATAGCCGATCAG-3'
T107H	sense	5'-CTGATCGGCTATACG**cac**ACGGTGCCGGATCC-3'
	anti-sense	5'-GGATCCGGCACCGT**gtg**CGTATAGCCGATCAG-3'
T107K	sense	5'-GATCGGCTATACG**AaG**ACGGTGCCGGATCC-3'
	anti-sense	5'-GGATCCGGCACCGT**CtT**CGTATAGCCGATC-3'
T107R	sense	5'-CTGATCGGCTATACG**cgc**ACGGTGCCGGATCC-3'
	anti-sense	5'-GGATCCGGCACCGT**gcg**CGTATAGCCGATCAG-3'

[a]Mismatch bases in lower case, mutated codon in boldface.

Table S17. Primers used for mutagenesis of the active site nucleophile

Enzyme	mutation	primer[a]	
*Ak*AS	C53S	sense	5'-GTCAATTCGGGCGTG**aGC**GGCCCGTCACGGATG-3'
		anti-sense	5'-CATCCGTGACGGGCC**GCt**CACGCCCGAATTGAC-3'
*Rp*AS	C53S	sense	5'-GTGCAGTCGACGATC**aGC**GGCCCGTCCCGGATG-3'
		anti-sense	5'-CATCCGGGACGGGCC**GCt**GATCGTCGACTGCAC-3'
*Sa*AS	C49S	sense	5'-GTGCAATCCCCCATC**aGC**GGCGCCAGCCGGATG-3'
		anti-sense	5'-CATCCGGCTGGCGCC**GCt**GATGGGGGATTGCAC-3'
*Sp*AS1	C53S	sense	5'-GTTCAGGCGACGGTA**aGC**GGGCCATCGCGCATG-3'
		anti-sense	5'-CATGCGCGATGGCCC**GCt**TACCGTCGCCTGAAC-3'
*Sp*AS2	C49S	sense	5'-ATCCAATCACCGATC**aGC**GGCTCGTCCCGGATG-3'
		anti-sense	5'-CATCCGGGACGAGCC**GCt**GATCGGTGATTGGAT-3'
*Ak*PMH	C52S	sense	5'-ACCCAGGCTGTACCT**aGC**GGACCAGGCCGAGCC-3'
		anti-sense	5'-GGCTCGGCCTGGTCC**GCt**AGGTACAGCCTGGGT-3'
*Bc*PMH	C57S	sense	5'-ACGACGTGCGTGCCG**TcT**GGTCCGGCAAGGGCA-3'
		anti-sense	5'-TGCCCTTGCCGGACC**AgA**CGGCACGCACGTCGT-3'
*Sp*PMH	C53S	sense	5'-TCCGTGACCAACCCC**aGC**GGGCCGTCGCGTGCC-3'
		anti-sense	5'-GGCACGCGACGGCCC**GCt**GGGGTTGGTCACGGA-3'

[a]Mismatch bases in lower case, mutated codon in boldface.

References

[1] S. Jonas, B. van Loo, M. Hyvonen, F. Hollfelder, *J Mol Biol* **2008**, *384*, 120–136.

[2] S. Jonas, Structural and Mechanistic Aspects of Catalytic Promiscuity in the Alkaline Phosphatase Superfamily., Trinity Hall, University of Cambridge, **2009**.

[3] B. van Loo, C. D. Bayer, G. Fischer, S. Jonas, E. Valkov, M. F. Mohamed, A. Vorobieva, C. Dutruel, M. Hyvönen, F. Hollfelder, *Catalytic Promiscuity and Evolution of Function in the Alkaline Phosphatase Superfamily: Phosphonate Monoester Hydrolases and Aryl Sulfatases*, **2017***, submitted*.

[4] A. Zamyatin, *Prog Biophys Mol Biol* **1972**, *24*, 107–123.
